# Molecular Evolution of MDM1, a “Duplication-Resistant” Gene in Vertebrates

**DOI:** 10.1371/journal.pone.0163229

**Published:** 2016-09-22

**Authors:** Monica R. Hensley, Rhys F. M. Chua, Yuk Fai Leung, Jer-Yen Yang, GuangJun Zhang

**Affiliations:** 1 Department of Comparative Pathobiology, Purdue University. West Lafayette, Indiana, United States of America; 2 Department of Biological Sciences, Purdue University. West Lafayette, Indiana, United States of America; 3 Purdue Institute for Integrative Neuroscience, Purdue University. West Lafayette, Indiana, United States of America; 4 Department of Basic Medical Sciences, Purdue University. West Lafayette, Indiana, United States of America; 5 Purdue University Center for Cancer Research. West Lafayette, Indiana, United States of America; 6 Purdue Institute for Inflammation, Immunology and Infectious Diseases (PI4D), Purdue University. West Lafayette, Indiana, United States of America; California State University Fullerton, UNITED STATES

## Abstract

**Background:**

The mouse double minute 1 (*Mdm1*) gene was first reported and cloned in mouse tumor cell lines as an oncogene candidate. Later, it was found that mutation of *Mdm1* might cause age-related retinal degeneration 2 in mice by genetic linkage analysis. Additionally, the MDM1 protein was found to be expressed in the centrosomes, cilia, and the nucleus of multiciliated tracheal epithelial cells in mice. These observations suggest that MDM1 may have some basal functions in cell physiology. However, the evolutionary history of this gene and its expression during embryonic development remain largely unexplored.

**Results:**

Using molecular phylogenetic analysis, we found that the *MDM1* gene encoded an evolutionarily conserved protein across all metazoans. We also found that the *MDM1* gene was in a conserved synteny in vertebrates. In almost all the species that were analyzed, there was only one *MDM1* gene based on current genome annotations. Since vertebrate genomes underwent two to three rounds of whole-genome duplications around the origin of the vertebrates, it is interesting that only one *MDM1* ohnolog was retained. This observation implies that other *MDM1* ohnologs were lost after the whole-genome duplications. Furthermore, using whole-mount *in situ* hybridization, we found that *mdm1* was expressed in the forebrain, nephric ducts, and tail buds during zebrafish early embryonic development.

**Conclusion:**

*MDM1* is an evolutionary conserved gene, and its homologous genes can be traced back to basal metazoan lineages. In vertebrates, the *MDM*1 gene is in a conserved synteny and there is only one *MDM1* ohnolog suggesting it is a “duplication-resistant” gene. Its expression patterns in early zebrafish embryos indicate that *mdm1* may play important roles in the development of the central nervous system, kidneys, and hematopoietic system.

## Background

Whole genome duplication (WGD) is a driving force of evolution across the phylogeny of eukaryotes [1]. In vertebrates, WGDs are important for the origin of vertebrate morphological novelties (such as endoskeleton, placodes, and neural crest) [2, 3]. Two successive rounds of WGD (1R and 2R) happened during the transition from invertebrate to vertebrate [1, 4, 5]. In addition, a third teleost-specific WGD occurred just before the separation of bony fish from lobe-finned fish [6–8]. These three WGDs have highly influenced the organization and the gene content of vertebrate genomes, speciation and development, since the functional and regulatory divergence of duplicated genes provides a genetic basis for morphological novelties [4, 9, 10].

After WGDs, the duplicated genes within a species become paralogs. Such paralogous genes that resulted from WGDs are called ohnologs in honor of Susumu Ohno, a founder of the genome-duplication hypothesis [11, 12]. Due to the vertebrate WGDs, singleton genes in non-vertebrate species will have 1 to 4 ohnologs in tetrapods, and 1 to 8 ohnologs in teleosts depending on the number of retained duplicates [13]. The fate of duplicated genes after WGD was generally explained by the DDC (duplication-degeneration-complementation) model [14]. Gene coding and regulatory sequence divergence lead to sub-functionalization (*i*.*e*. functional split) and neo-functionalization (*i*.*e*. new function). The function redundancy of duplicates may also lead to non-functionalization (*i*.*e*. gene loss). Sub-functionalization and neo-functionalization contribute to the expansion of genetic tool kits and thus morphological novelties and diversities [15], whereas non-functionalization is a passive process. Recently, we start to realize that non-functionalization may also contribute to genetic variation and evolution through non-random gene loss, as suggested by certain genes found to be duplication-resistant [16, 17].

Mouse double minute (MDM) genes are a group of genes discovered on the highly amplified double minute, a small fragment of extra chromosomal DNA that is usually a circular DNA molecule without a centromere or telomere in transformed mouse 3T3DM cells [18]. Originally, four genes, *MDM1-MDM4*, were named according to early research in the mouse double minutes. However, *MDM3* is no longer a valid gene according to current genome annotations. *MDM2* and *MDM4* are ohnologs from WGDs and they have been well studied for their roles in regulating TP53 through ubiquitination [19, 20]. In contrast, *MDM1* has been much less studied since its discovery as a nuclear protein and was found to fail to transform the mouse 3T3 cells [18, 21]. It is known that there are at least four alternatively spliced transcript variants for *Mdm1* in mice [22], and the 3.1kb mRNA is expressed in a variety of adult mouse tissues with the highest expression in testis, suggesting that it may have a role in spermatogenesis. Immunohistological staining revealed that the mouse MDM1 protein translated from the 3.1kb mRNA is located in the nuclei, not nucleoli, evidenced by speckled or punctate patterns of staining, while other sized MDM1 protein isoforms coded from the same gene are located in the cytoplasm [21]. Later, the same group tested the tumorigenic roles of both *Mdm1* and *Mdm2* in NIH3T3 and Rat2 cells by overexpressing full length genomic DNA in a cosmid vector. Murine cell lines with *MDM1* overexpression did not form tumors in athymic nude mice by subcutaneous xenograft, while xenografted *MDM2* overexpression stable cell lines did form tumors in 100% of xenografts [18]. In addition to tumorigenesis, *Mdm1* has also been associated with age-related retinal degeneration 2 (arrd2) in mice through positional cloning [22]. Upon further examination of the *Mdm1* transcripts, it was revealed that a retina specific *Mdm1* transcript was mutated (non-sense) in exon 8, which is 960 bps upstream of the original stop codon. This mutation led to the retinal *Mdm1* transcript nonsense-mediated decay in arrd2 mice. However, there was no association of the *MDM1* locus with age-related macular degeneration in human patients [22]. Recently, another study looking at the transcriptional profiles of mouse multiciliated tracheal epithelial cells found high levels of *Mdm1* gene expression. MDM1-GFP protein was found localized to centrosome and primary cilia in 293T cells after transient transfection, suggesting that MDM1 might have cellular functions related to cilia [23]. Very recently, MDM1 was reported to be a microtubule-binding protein and a negative regulator for centriole duplication [24].

Aside from the above knowledge on *MDM1*, we know nothing about *MDM1* gene expression patterns during vertebrate development or its evolutionary history. Here, we first analyzed the MDM1 evolutionary history using molecular phylogenetic and syntenic relationships analyses. Then, we examined *mdm1* gene expression patterns during early embryonic development in zebrafish. We found that MDM1 is a “duplication-resistant” gene in vertebrates. Its gene expression patterns suggested it may participate in or regulate early development of brain, notochord and kidneys in zebrafish.

## Methods and Materials

### Zebrafish strains and husbandry

All zebrafish were raised and maintained at the AAALAC-approved animal housing facilities according to protocols approved by the Purdue Animal Care and Use Committee (PACUC Protocol # 1210000750). The wild type line used in this study is from the TAB background, which was created at Hopkins Lab at MIT (http://zfin.org/ZDB-GENO-010924-10). Zebrafish embryos were raised in fish system water and incubated at 28°C. Day 1 to day 3 old embryos were collected and fixed in 4% PFA (paraformaldehyde) for *in situ* hybridization. All protocols for experiments were approved by Purdue University institutional review board (IRB protocol # 12–029).

### Gene sequence retrieval, phylogenetic, and syntenic analysis

Sequences of human and zebrafish were identified through searches of the NCBI database using the gene symbol for MDM1. The remaining MDM1 protein sequences were identified through BLASTp in Ensembl and NCBI using the human and zebrafish MDM1 sequences as queries with default settings [25, 26]. Blink (BLAST Link) in NCBI was employed to identify/verify the MDM1 in closely related taxa from the pre-computed sequence alignments. Representative MDM1 sequences from the major metazoan taxa were selected based on their taxonomic positions. Whenever there were multiple isoforms, the longest sequence was chosen for analysis. The final MDM1 protein accession numbers and species are listed in the S1 Table.

The multiple sequence alignments were generated with the MUSCLE program [27], and can be found in the S1 File. Phylogenetic analyses using the multiple protein alignments were conducted with Baysian analysis (BP), Maximum Likelihood (ML) and Neighbor Joining (NJ) methods [28–30]. Best fitting evolutionary models were tested in the MEGA 6.06 program using maximum likelihood and default parameters [30]. The models with the lowest BIC scores (Bayesian Information Criterion) are considered to describe the substitution pattern the best, and in our studies we chose JTT+G (α = 2.087). For BP phylogenetic analysis, 10 million generations were run using the following parameters with MrBayes 3.2.6: nruns = 2, nchains = 4, aamodel = fixed (Jones), rates = gamma ngammacat = 8, samplefreq = 500, burninfrac = 0.25. For ML phylogenetic analysis, PHYML 3.1 [29] was chosen using the following parameters: datatype = AA; model = JTT, gamma = yes, gamma distribution parameter = estimate (α = 1.997); bootstrap = 1000. NJ analysis was carried out using MEGA 6.06 with the following parameters: Test of phylogeny = bootstrap method, No. of Bootstrap Replications = 10,000, Substitution Type = Amino Acid, Model = JTT, Rates among sites = Gamma Distribution (G), Gamma Parameter = 2.087, Gaps/Missing Data Treatment = Pairwise and/or complete deletion. BP and ML phylogenetic trees were visualized and displayed with FigTree V1.4.2 (http://tree.bio.ed.ac.uk/software/figtree).

Selected vertebrate *MDM1* genomic structure and synteny analysis were performed using the UCSC genome browser and Ensembl database: elephant shark (*Callorhinchus milii)* calMil1; zebrafish (*Danio rerio)* GRCz10; chicken (*Gallus gallus)* Galgal4; human (*Homo sapiens)* GRCh38.p3; spotted gar (*Lepisosteus oculatus)* LepOcu1; mouse (*Mus musculus)* GRCm38.p4; Western clawed frog (*Xenopus tropicalis)* JGI 4.2; platyfih (*Xiphophorus maculatus*) Xipmac4.4.2; Japanese medaka (*Oryzias latipes)* HdrR.

### Gene cloning, *in situ* hybridization and imaging

The full-length coding region of the zebrafish *mdm1* genes was amplified by RT-PCR. Embryos of 1–3 days post fertilization (dpf) were mixed for total RNA isolation using TRizol reagent (Thermo Scientific) and reverse transcriptions were performed using SuperScript® III First-Strand Synthesis System (Thermo Scientific) following the manufactory instructions. Phusion® High-Fidelity DNA Polymerase master mix (New England Biolabs) was used for PCR amplification. PCR primers used for zebrafish *mdm1* gene: Forward primer, 5’ATGCCTGTCCGTTTCAAGGGAATCA3’ and reverse primers 5’GCTTTTTCCCCAAAATTCCTGTTTTCGT3’. The PCR products were purified using GeneJET Gel Extraction Kit (Thermo Scientific) before cloning into pJet1.2 vector using the CloneJET PCR Cloning Kit (Thermo Scientific) according to the user manual. Orientations of gene inserts were verified by Sanger sequencing and endonuclease diagnosis. Sense and antisense *mdm1* riboprobes were synthesized by *in vitro* transcription using T7 DNA polymerase (NEB) with Xba I linearized plasmid constructs. Riboprobes were purified by SigmaSpin Post-Reaction Clean-Up columns (Sigma, S5059) and stored at -80°C before use.

Whole mount *in situ* hybridization (WISH) was carried out according to previous published methods with some modifications [31, 32]. Briefly, zebrafish embryos were collected, maintained and staged according to the zebrafish development staging guide [33]. Chorions were removed using pronase treatment before fixation. Zebrafish embryos were then fixed with 4% PFA overnight at 4°C and then dehydrated using serial methanol (25%, 50%, 75% and 100%) with PBST (Phosphate-buffered saline solution with 0.1% tween-20). Dehydrated embryos were stored at -20°C until used for experiments. To perform the WISH, fish embryos were rehydrated using the reverse gradient methanol in PBST. Embryos were then bleached with 5% H_2_O_2_ in PBT until all the pigmentation on the fish embryos were not visible. Permeabilization was carried out using proteinase K 10 μg/ml in PBT: 8–18 hours embryos, no treatment; 24 hours embryos, 5 minutes; 48 hours embryos, 30 minutes at room temperature. Embryos were then fixed in 4% PFA with 0.2% gluteraldehyde for 30 minutes at room temperature. After washing out fixatives with PBT (3 x 10mintes), embryos were incubated in pre-hybridization solution [50% formamide, 5XSSC (0.75 M NaCl, 75mM sodium citrate, pH 7.0.), 2% Roche blocking powder, 0.1% Triton-X, 50 mg/ml heparin, 1 mg/ml Torula yeast RNA, 1 mM EDTA, 0.1% CHAPS, DEPC-treated ddH_2_O] overnight at 65°C in hybridization oven with gentle shaking (60 rpm). Riboprobes were added at the second day then embryos were further hybridized for another 48hours before washing out unbound riboprobes using 2X SSC and 0.2X SSC (3 x 30min for each solution). Then embryos were washed with KTBT (50 mM Tris-HCl, pH 7.5, 150 mM NaCl, 10 mM KCl, 0.3% Triton-X) before antigen blocking using 20% sheep serum in KTBT for 3 hours. Anti-digoxygenin antibody conjugated to alkaline phosphatase (Roche) was added into the blocking solution in a ratio of 1:5000 and embryos were incubated overnight at 4°C with gentle shaking. After 6 time 1-hour washing with KTBT in the following day, the color reaction was carried out in NTMT solution (100 mM Tris-HCl, pH 9.5, 50 mM MgCl2, 100 mM NaCl, 0.1% Triton-X, 1 mM levamisole) with 75 mg/ml NBT (Roche), 50 mg/ml BCIP (Roche), and 10% DMF (N,N-dimethylformamide). Color development was monitor closed and stopped with NTMT washing when proper color density was achieved. For histological analysis, post-hybridization embryos were equilibrated in 15% sucrose then 30% sucrose in 20% gelatin, after which they were embedded in 20% gelatin for cryosectioning (10–12 μm). Images were acquired using AxioCam MRc camera on Zeiss Stereo Discovery.V12 and Axio Imager 2 compound microscope.

## Results

### MDM1 proteins form a distinct protein family

As our current knowledge of MDM1 is very limited across species, we first searched for conservation of this gene using the BLASTp program. Using human MDM1 protein sequence as a query, we found that the human MDM1 protein only shares similarities with MDM1 proteins from other species of metazoans, and it has no similarity with MDM2 and MDM4 proteins even though they bear similar names. To assess the MDM1 protein diversities and whether there are other domains in MDM1 proteins, conserved domain analysis was performed using the definition from the Pfam database which revealed that MDM1 proteins from different metazoan species form a distinct gene family (pfam15501).

### Molecular evolutionary history of MDM1 proteins

**Fig 1:**
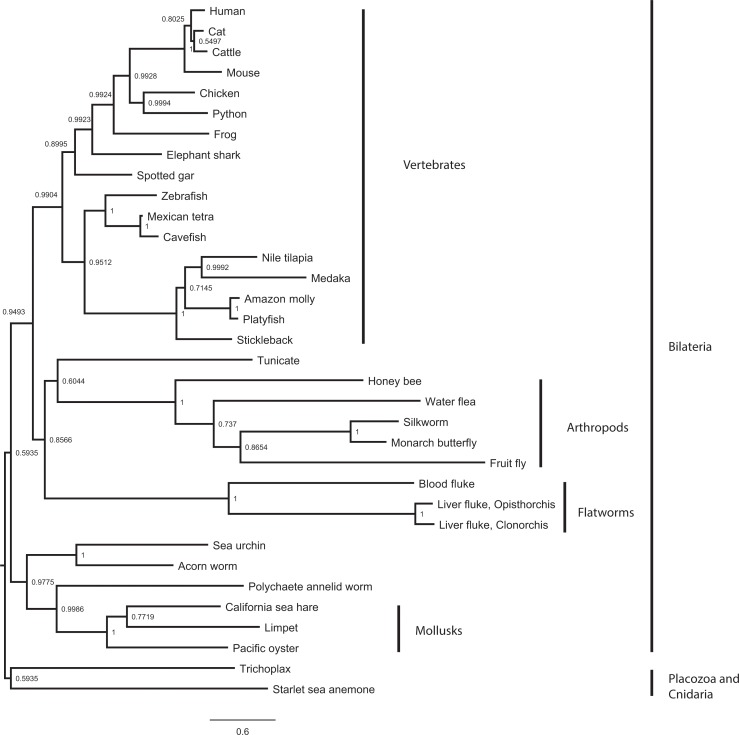
Extended majority-rule consensus tree for the Bayesian phylogenetic analysis of MDM1 proteins. Numbers at each node indicate posterior probability (pp) values based on ten million replicates. Branch lengths are proportional to means of the pp densities for their expected replacements per site.

To understand the evolutionary history of the *MDM1* genes, we performed molecular phylogeny analysis using BP, ML and NJ methods. In general, BP and ML phylogenies are more similar (except the position of the sea anemone) than NJ (Fig 1, S1 and S2 Figs). In all these phylogenies, the vertebrate species formed a distinct clade, whereas those from invertebrate species formed other clads. The invertebrate phylogenetic relationships cannot be solved clearly in all three phylogenies (reflected by lower posterior probability/likelihood/bootstrap/values) due to the divergence of MDM1 protein sequence. However, the arthropods, mollusks and flatworms were grouped together indicating that their MDM1 protein sequences are similar to each other within each group. The overall phylogenetic pattern from the Bayesian analysis was close to the current consent relationships of the metazoan systematics [34] (Fig 1), but still did not mimic metazoan taxonomy. This is not surprising since not all genes are suitable for molecular systematics. From our analyses, this MDM1 protein family evolved early in metazoa, as it was already present in both the trichoplax and cnidarian, the basal lineages of metazoa. The early origin of MDM1 suggests that MDM1 potentially has important basal functions for cell physiology in multicellular organisms. Our BLASTp search shows all vertebrates have only one MDM1, except for a few partially annotated sequences (or pseudogenes) in wild pig (*Sus scrofa*) and zebra finch (*Taeniopygia guttata*). Since BLASTp only picks up active genes and not pseudogenes or deleted genes, and since all vertebrate genomes underwent 2–3 rounds of WGDs [1, 5], our BLASTp result suggests that other MDM1 ohnologs underwent gene non-functionalization.

### Conserved *MDM1* synteny in vertebrates

**Fig 2:**
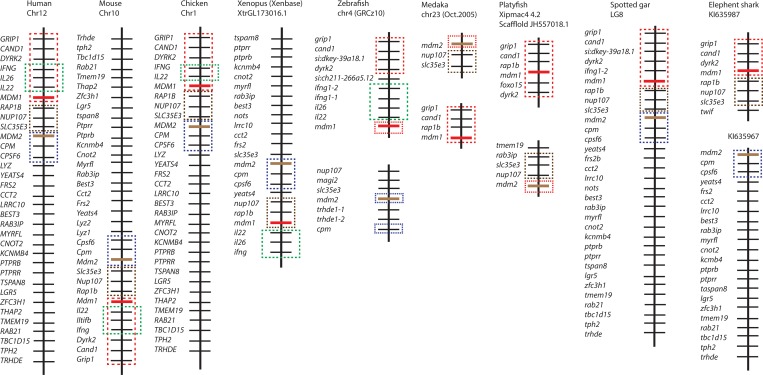
The synteny of *MDM1* in nine representative vertebrate species. The illustration of the gene and their sizes are not proportional to the length of the bars. *MDM1* is highlighted in red, and *MDM2* is highlighted in brown. *MDM1* synteny (*MDM1*- *DYRK2*-*CAND1*-*GRIP1*) is boxed with red lines. *MDM2* synteny (*MDM2*-*CPM*-*CPSF61*) is boxed with blue lines. *RAP1b* synteny (*RAP1B*-*NUP107*-*SLC35E3*) is boxed with brown lines. *IL22* synteny (*IL22*-*IL26*-*IFNG*) is boxed with green lines.

According to our phylogenetic analysis, *MDM1* is a conserved gene across many vertebrate species. However, phylogenetic analysis alone may not be sufficient for determining the gene orthologous relationships, especially in the situation of gene duplications followed by reciprocal gene losses between organisms or rapid lineage-specific gene expansions [12]. One example is the reciprocal loss of FGF D family members in different vertebrates [15]. To further explore the evolutionary history of the vertebrate *MDM1* gene, we examined and compared its chromosome locations in different vertebrate species. The *MDM1* orthologs are in a conserved synteny in all examined vertebrate species (Fig 2). Four genes (*GRIP1*, *CAND1*, *DYRK2* and *MDM1*) are linked together on the chromosome from elephant shark to human. Among these 4 genes, *DYRK2* has been known to have two ohnologs, *DYRK2* and *DYRK4* in mammals [35], supporting the loss of other *MDM1* ohnologs in vertebrates. In the tetrapod, zebrafish, and spotted gar genomes, but not medaka, platyfish, and elephant shark, this MDM1 synteny is next to another two syntenies that usually stay together on the same chromosome: *rap1b*-*nup107-slc35e3*, and *cpsf6*-*cpm-mdm2* (Fig 2). A fourth synteny, *IFNG-IL26-IL22*, started to be found next to *MDM1* synteny in the tetrapod lineage. Our linkage analysis therefore reveals that *MDM1* is in an evolutionary conserved synteny in vertebrates, even though there has been extensive reshuffling of gene position during evolution.

### Gene expression of *mdm1* in zebrafish

**Fig 3:**
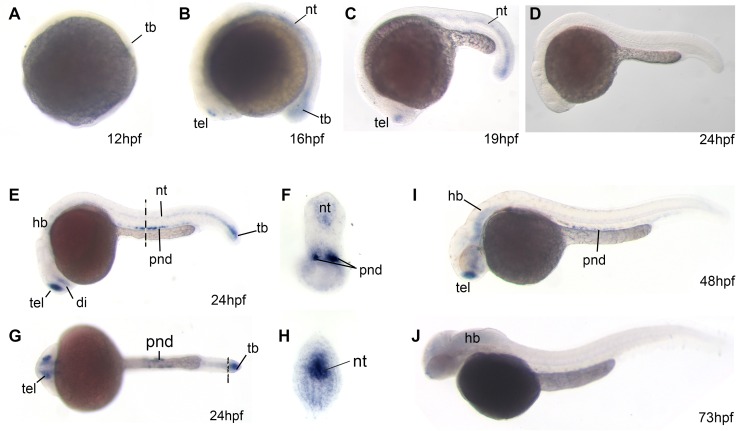
Zebrafish *mdm1* whole mount *in situ* hybridization gene expression patterns during early development. **A.** At 12 hpf (hours post fertilization), *mdm1* begins to be expressed at the forming tail bud (tb). **B.** At 16hpf, *mdm1* expression expands to the telencephalons (tel) and neural tube (nt). **C.** At 19hpf, *mdm1* expressed similarly with 16hpf except the expression in notochord reduced. **D.** mdm1 sense riboprobe control. **E.** At 24hpf, *mdm1* is expressed at the perinephric duct (pnd) in addition to the continued expression in the telencephalons (tel), diencephalons (di), and tail bud (tb) **F.** 24hpf, transverse section showed that *mdm1* is expressed at the perinephric duct (pnd) region and neural tube (nt). Dashed line indicates the position of the transverse section of **E**. **G.** Ventral view of panel **E.** Dashed line indicates the position of the transverse section of **H. H.** Transverse section through the tail bud (tb) of 24hpf embryo. **I.** 48hpf *mdm1* gene expression expands into the hindbrain (hb). **J**. 72hpf *mdm1* is dominantly expressed in the hind brain (hb).

Although there have been some experiments performed at the cellular level looking at *Mdm1* mRNA in different tissues by Northern blots [21], the roles of the evolutionarily conserved *MDM1* gene have not yet been studied during animal development. Since zebrafish is such a great model for studying vertebrate development and human disease [36], we decided to elucidate the gene expression patterns of *mdm1* during the early developmental stages of zebrafish using *in situ* hybridization. As expected, zebrafish *mdm1* is already expressed at 50% epiboly, ~12 hpf, and is dominantly expressed in the tail bud (tb) region (Fig 3A). At 16 hpf and 19 hpf, *mdm1* expression expands to the neural tube (nt) and forebrain (fb), in addition to expression in the tail bud (Fig 3B and 3C). Starting at 24 hpf through 36hpf *mdm1* expression is found in the pronephric duct (pnd) (Fig 3E–3H). Between 24hpf to 36hpf, *mdm1* expression in the neural tube is restricted to hind brain (hb) (Fig 3I and 3J). These dynamic expression domains of the *mdm1* gene suggest that Mdm1 may have a role in participating or regulating organ development.

## Discussion

Two rounds of consecutive WGDs (1R and 2R) occurred in the basal vertebrate lineage, and a 3R WGD (3R) occurred in the teleost lineage. These WGDs resulted in the expansion of gene numbers, reorganization of vertebrate genomes, and an increase of genetic interaction complexity [4, 9, 10]. Interestingly, certain genes are “resistant” (duplicates were lost quickly) to these WGDs as they are present as a single ohnolog in almost all the species. The *MDM1* gene was discovered as a candidate oncogene in mouse 3T3DM cells, however the evolutionary history of this gene and its developmental functions remain largely unknown. In this report, we first analyzed the protein domains of the MDM1 family, and found that MDM1 proteins form a distinct protein family in the metazoan lineage. The *mdm1* gene is also widely expressed during early zebrafish development, suggesting that it may play important roles in embryogenesis.

MDM1 forms a distinct protein family whose molecular and cellular functions remain largely unknown. The *MDM1* gene family should not be confused with the *MDM2* gene family, which is a well-known E3 ubiquitin ligase and is important to mediate the degradation of various tumor suppressers such as TP53, FOXO3a and CDH1 [37, 38]. Consistent with this, we did not find any sequence similarities between MDM1 and MDM2/MDM4. Our molecular phylogenetic analysis revealed that the MDM1 family arose in the basal lineage of metazoa. The phylogeny of MDM1 approximately agrees with current taxonomies of the metazoa [34]. The elephant shark MDM1 was grouped more closely to tetrapods than teleosts. This phenomenon was also reported with 149 other shark genes [39]. One possible reason might be that MDM1’s differential evolving rates in different species. Alternatively, it could be caused by the incompletely solved phylogenic relationship due to the relatively low likelihood/bootstrap values of spotted gar and elephant shark branches (S1 and S2 Figs). Another explanation could be different ohnologs being deleted (ohnolog-gone-missing) in different species, since phylogeny potentially loses its power to deal with gene duplication and reciprocal gene loss in different organisms [12, 15]. Our *MDM1* synteny analysis supported the later possibility because the gene content around the *MDM1* locus in elephant shark is more similar to those in tetrapods. The interrelationship among invertebrates is inconsistent in our phylogenies, even though anthropods, flatworms, and mollusks formed distinct clads. This inconsistency is likely caused by the sequence divergence in invertebrate MDM1 protein, a phenomenon that has been observed in the phylogeny of certain genes. As a result, their phylogenies may not mimic the taxonomy of the corresponding animals.

Due to the WGDs in vertebrates, four or eight ohnologs are expected in each species if there are no gene losses in tetrapods and teleosts. For example, there are three Hedgehog homologous genes, *SHH*, *IHH*, and *DHH* in most of the vertebrates, while there is only one Hedgehog gene in invertebrates. Another example is the *HOX* gene clusters. There are four clusters of *HOX* genes in tetrapods and seven to eight clusters in teleosts [6]. In contrast, according to the current genome annotation, only one *MDM1* is present in the vertebrate species we analyzed, suggesting its ohnologs were lost after the WGDs. Only a few cases, such as the wild pig and zebra finch have more than one MDM1 fragments that are likely pseudogene or artifacts of incomplete genome annotations. In general, the gene loss/non-functionalization could be mediated by pseudogenization or gene locus deletion due to functional restriction or genetic random drift [12, 15]. Independent gene loss in each vertebrate taxon involves many gene-loss events, a very unlikely scenario based on the principle of parsimony. Therefore, the more likely scenario is that MDM1 was lost in basal vertebrates shortly after WGDs.

This kind of “duplication-resistance” may be explained by the less-is-more hypothesis that is related to the gene’s function and dosage sensitivity [25, 26]. For example, it was found that genes expressed in early development are more duplication-resistant than the genes expressed later [27]. Indeed, this is the case of zebrafish *mdm1*, as it is expressed in early development (Fig 3). Similarly, dosage sensitive genes are more resistant to duplication [40, 41]. *MDM1* DNA copy number was found amplified in many human cancers and zebrafish tumors [21, 42], suggesting that the increase of *MDM1* dosage might be deleterious. Along this line, MDM1’s *in vivo* oncogenic function requires further experimental investigations in animal models although the early mouse *Mdm1* overexpression experiment failed to transform NIH3T3 and Rat2 cell lines in mouse xenografts [18].

**Fig 4:**
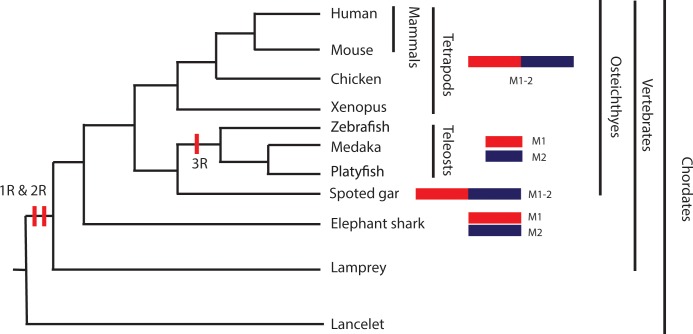
The scenario of *MDM1* evolution from the syntenic analysis in vertebrates. The vertebrate phylogenetic relationships are adopted from the references [47, 48]. In tetrapod and spotted gar, the *MDM1* and *MDM2* syntenies (M1 and M2) are neighbors next to each other, while the two syntenies are located on different chromosomes in elephant shark and teleosts. The vertical red bars on the tree indicate the whole genome duplication events. There are two rounds of WGDs (1R and 2R) before the origin of the vertebrates and a third round happed before the separation of the teleosts (3R).

Our syntenic analysis revealed that the vertebrate *MDM1* genes are located in an evolutionarily conserved synteny, which is composed of the four core genes (*GRIP1*, *CAND1*, *RAP1* and *MDM1*). The *MDM1* synteny is already formed in the elephant shark, and it is next to *MDM2* synteny (*cpsf6*-*cpm-mdm2*) in the basal bony fish, spotted gar whose genome did not undergo teleost specific WGD [43], and they remain as a synteny in the genomes of tetrapods. However, the *MDM1* and *MDM2* syntenies are located on different chromosomes within bony fish species (Fig 2). Based on the phylogeny of MDM1 and events of WGDs in vertebrates, we propose a scenario for the evolution of linkage alterations between *MDM1* and *MDM2* syntenies (Fig 4): *MDM1* and *MDM2* genes might be located in one synteny (M1-2) in the vertebrate ancestor. The synteny was broken into two separate smaller syntenies (M1 and M2) in chondrichthyans but not in Holostei. The M1 and M2 became separated syntenies in teleosts after whole-genome duplications (3R) in bony fish; whereas they remained stable as one synteny (M1-2) in the tetrapod lineage. Contrary to the single *MDM1* ohnolog gene in analyzed vertebrates, its neighboring genes DRYK2 and MDM2 both have other ohnologs in vertebrates [19, 20, 35], supporting our idea that *MDM1* is a “duplication-resistant” gene. *MDM1* synteny was already noticed to be linked with a synteny of immunity-related genes (*INFG1*, *IL22 and IL26*) [44]. The neighborhood of *IFNG* synteny and *MDM1* synteny was already established in spotted gar, before the separation of teleost lineage. The two syntenies are next to each other on the same chromosome in zebrafish, but not in medaka and platyfish, suggesting the gene position reshuffle occurred in teleost lineage after 3R WGD. *IFNG* synteny remains stable since the separation of tetrapods. Although *IFNG* synteny is not next to *MDM1* synteny in elephant shark, they might be in other shark species. Future syntenic analysis in other chondrichthyans will be informative since it is not known whether elephant shark is a derivative or not.

We have found that *mdm1* is dynamically expressed in the forebrain (telencephalon and diencephalon), notochord, tailbud and pronephral ducts, suggesting it is involved in their organogenesis. The limited expression in the notochord and tailbud suggest zebrafish *mdm1* may be only required transiently for their normal development. For the majority of development-related genes, the mRNA expression domains are generally consistent with protein expression domains. But for the secreted proteins such as morphogen (e.g. SHH, BMP, WNT) and cytokines, their mRNA and protein domains may slightly vary, because the mRNA is usually limited to cells that produce the mRNAs and proteins. However, this situation is not applied to MDM1, because MDM1 is mainly a nuclear protein that is not secreted [21]. *Mdm1* has been shown to located in cilia and the centriole [23], which suggest that it might interact with members of the Hedgehog signaling pathway, as this pathway is tightly linked with cilia [45]. Interestingly, the regions in early zebrafish embryos that express *mdm1* also express Hedgehog signaling member genes, such as *shha* [46]. Further functional studies will be necessary to elucidate its roles during development and whether there is a genetic interaction between Hedgehog signaling and *mdm1*.

## Supporting Information

S1 Fig Molecular phylogenetic tree generated using ML analysis on MDM1 proteins as obtained with JTT plus gamma distribution. Numbers at each node denote bootstrap values above 50% based on 1,000 replicates. Branch lengths are proportional to expected replacements per site. The tree is rooted with the trichoplax sequence.
(EPS)

S2 Fig Neighbor joining phylogeny for MDM1 as obtained with JTT plus gamma (α = 2.087). Bootstrap scores based on 10,000 replicates for each node were labeled around each node. This tree is rooted using the trichoplax MDM1 as an outgroup.
(EPS)

S1 File MDM1 multiple sequence alignment generated by MUSCLE. (PDF)

S1 Table Protein sequences and species used in this analysis. (DOCX)
