# Supplementary material for: Molecular Evolution of MDM1, a “Duplication-Resistant” Gene in Vertebrates
Source: PLoS One. 2016 Sep 22;11(9):e0163229. doi: 10.1371/journal.pone.0163229 (PMC5033493; doi:10.1371/journal.pone.0163229)
Supplement: S1 File — (PDF) [file pone.0163229.s003.pdf]

```
                            10        20        30        40        50        60        70        80        90       100
                   ....|....|....|....|....|....|....|....|....|....|....|....|....|....|....|....|....|....|....|....|
daphnia            ----------------------------------------------------------------------------------------------------
silkworm           ----------------------------------------------------------------------------------------------------
butterfly          ----------------------------------------------------------------------------------------------------
bee                ----------------------------------------------------------------------------------------------------
fly                ----------------------------------------------------------------------------------------------------
mansoni            ----------------------------------------------------------------------------------------------------
viverrini          ----------------------------------------------------------------------------------------------------
sinensis           ----------------------------------------------------------------------------------------------------
anemone            ----------------------------------------------------------------------------------------------------
Trichoplax         ----------------------------------------------------------------------------------------------------
urchin             ----------------------------------------------------------------------------------------------------
Saccoglossus       ----------------------------------------------------------------------------------------------------
ciona              ----------------------------------------------------------------------------------------------------
telata             MIHEWHVRQCKVIEKIIKYTLTEYLESNNRYPQNPFRVMTQFVYYASSWLGKLKNLRNICEIKSRLQITFNIHCTQKQVATNSQNLADDLIADIIVLINR
Medaka             ----------------------------------------------------------------------------------------------------
tilapia            ----------------------------------------------------------------------------------------------------
stickleback        ----------------------------------------------------------------------------------------------------
molly              ----------------------------------------------------------------------------------------------------
platyfish          ----------------------------------------------------------------------------------------------------
mouse              ----------------------------------------------------------------------------------------------------
human              ----------------------------------------------------------------------------------------------------
cat                ----------------------------------------------------------------------------------------------------
cattle             ----------------------------------------------------------------------------------------------------
python             ----------------------------------------------------------------------------------------------------
chick              ----------------------------------------------------------------------------------------------------
xenopus            ----------------------------------------------------------------------------------------------------
shark              ----------------------------------------------------------------------------------------------------
gar                ----------------------------------------------------------------------------------------------------
zebrafish          ----------------------------------------------------------------------------------------------------
tetra              ----------------------------------------------------------------------------------------------------
cavefish           ----------------------------------------------------------------------------------------------------
limpet             ----------------------------------------------------------------------------------------------------
oyster             ----------------------------------------------------------------------------------------------------
aplysia            ----------------------------------------------------------------------------------------------------
Clustal Consensus

                           110       120       130       140       150       160       170       180       190       200
                   ....|....|....|....|....|....|....|....|....|....|....|....|....|....|....|....|....|....|....|....|
daphnia            --------------------MQ------NQLYLQ---VLRRSQSEGPHQR--AGGAGPGGAQGG----VAAC----------------------EPGPMA
silkworm           --------------------MERSILA-ESRHLR------WASELHSEYR-------STYRWHE----FKEQ----------------------QQGVVK
butterfly          -----------------MLVISLHQLL-HSEYRS---TYRWHEYKEQQQQ--QGVVKQPAPTPP----TALP----------------------IARGAI
bee                --------------------MPANKGN-LS------------------------TPHKEKSGNG----LTTG----------------------ASKKKK
fly                ------MIGSFWNLCRACPSMPVD-KL-HSEYRS---TYRWH--EFTGNS--RPEVVRRAPAPN-----PSQ----------------------FVGATN
mansoni            ---------MGKITEEPLFQKKRKFEL-TNEFNL---NTQFANPKEDDYFVIGPSDGKFSNSIA----YRNK----------------------MKRNAR
viverrini          ---------------------MVQATD-ETLYQQGQGFNKSKEHSLGRYY--SDPNQDDYFIIG-----PTD----------------------KF----
sinensis           ---------------------MVQTTE-KTLFQQRPHFNKENEHSLGGHY--SDPTQDDYFIIG-----PTD--------------------------KF
anemone            --------------------MHRKCQY-ESEYHA---NF------------------------------KPF----------------------EVQLNK
```

```
Trichoplax          --------------------MPVRFENQAANFQD-------------------RPTVEKSSTAG----IPSE----------------------ESSTAR
urchin              --------------------MPVRAKS-SSEYVK---KFRWNDSVKSASF--TPTKEQNAPQAGMSSVGHAD----------------------SFAITR
Saccoglossus        ----------------------------------------------------------------------------------------------------
ciona               --------------------MPVKFRG-ASEYTS---TYKWSPSYRSAEF--QPTEQQLAVEAG----LRSD----------------------EMGLLM
telata              NSMIVETNLKDCHMLHIFTIAYCHTTG-RTEYNS---QF-----KRKQAS--KNSKNHTSRVAG----VASI----------------------DIGDIN
Medaka              --------------------MTVRFKS-QTEYQR---SF-----TLPRPS--GARALACDPAAH----PSSD----------------------PTRNTF
tilapia             --------------------MTVRFKS-QTEYQK---SF-----GVSRSR--SASPQRCMPLAG----LRSD----------------------QSGVSR
stickleback         --------------------MTARFKC-RSEYQK---SF-----RVPRST--SVCPQRGAPPAG----LRCD----------------------QMGICR
molly               --------------------MTVRFKS-QSEYQK---SY-----GVPRSR--SVSPQRCRPLAG----LRPD----------------------QNRISR
platyfish           --------------------MTVRFKS-QSEYQK---SY-----GVPRSR--SVSPQRCRPLAG----FRPD----------------------QNRISR
mouse               --------------------MPVRFKG-LSEYQR---NFLWKKSYLSESY--NPSVGQKYSWAG----LRSD----------------------QLGITK
human               --------------------MPVRFKG-LSEYQR---NFLWKKSYLSESC--NSSVGRKYPWAG----LRSD----------------------QLGITK
cat                 --------------------MPVRFKG-LSEYQR---NFLWKKSYLSESY--NPSVGRRYPWAG----LRSD----------------------QLGITK
cattle              ------------------------LQG-LSEYQR---NFLWKKSYLSESC--NSSMGRRHPWAG----LRSD----------------------QLGITR
python              --------------------MPVRWKG-LSEYKR---NFKWRQSDQSRLC--SSTHEQKCRWAG----LRSD----------------------EFGISR
chick               --------------------MPVRFRG-LSEYKR---NFRW---KTPELC--SPSQEQKSPWAG----LRSD----------------------QLGITR
xenopus             --------------------MPVQFKG-LSEYDR---NYTWKNHEDLEQ------EPTCSRWAG----LRSD----------------------ELGISK
shark               ---------------------PVHFRG-LSEYKR---NFKWNDSKRSRSC--SPSPEQKALWAG----LPSE----------------------QLGNTR
gar                 ------------------MKMPVRFKG-VTEYKK---RYGWKDSGSP------VSPDLRAPGAG----LRSD----------------------QLGITK
zebrafish           --------------------MPVRFKG-ISEYRS---KY---KGRTSRSR--SDSPHRRMRLAG----LRSD----------------------QSGITQ
tetra               --------------------M-------LCLYVF------------------------------------------------------------------
cavefish            --------------------MTVRFKG-ITEYQN---KY---KARARRVK--SAPPQRRMRLAG----VRSD----------------------QLGISR
limpet              -------------------MMPVRFKG-KSEYDT---HYKWVESYRGKNF--KPSLEQAAPQAGGTSEYKEQYKPFDNMLVEMNGKTHNTALDIPQQKGM
oyster              --------------------MFIQVQG-KSEYDR---NYKWLDSFKSKSF--TPTIEQIAPAAG----CESI----------------------ELGTVM
aplysia             --------------------MPVHFKG-ASEYKV---HYKWMDSFRSKSF--QPSQEQAARRAG----QISV----------------------NSSLTV
Clustal Consensus

                              210       220       230       240       250       260       270       280       290       300
                    ....|....|....|....|....|....|....|....|....|....|....|....|....|....|....|....|....|....|....|....|
daphnia             AWVHGRRRHV---------------------------TEQQRPEHWLVSA--------------------------------------------------
silkworm            QPAPTPPSALPIARGPIE-------------------PAIPRRKKYPGVA-----------------------YKTNELFDPVPADG-------------
butterfly           EPAMPRRKKYPGVA-----------------------YKTNELFDPAPAD--------------------------------------------------
bee                 RCTVKHKRHCKQYLQLH--------------------SEYRSTYTWHEYT----------------------GPHQEQVVRRAPQPP-------------
fly                 EPPLPRRKKCPEL------------------------AYKSHEFIIGSEY--------------------------------------------------
mansoni             SVSPKKKQENIGNG-----------------------LKQQSSAQINRQI-----------------------RKSSPILKPYRDEA-------------
viverrini           APSIAYRNRLKYRRRSASLRRQDSPGNPPN-------QQIPGNYLKKLKI-----------------------DRGNQKRHHKRENI-------------
sinensis            APSIAYRNRLKYRRRS---------------------ASLGRQDSLGHPS--------------------NQQGPGNNLKKLKIDHG-------------
anemone             EPKFSPAK-----------------------------PKVQET------------------------------KVAETLHKSSHNTH-------------
Trichoplax          EPPLQRKKRVTIQH-----------------------PDSAGESLKYQEA-------------------------NNKINQARPYTA-------------
urchin              EPPLQRRRKPIQEDNKM--------------------TYTTKQFMAAGDS-----------------------EDSSDIGKPNKIPG-------------
Saccoglossus        ----------------------------------------------------------------------------------------------------
ciona               EPTFTRKRRVMHRE-----------------------PEAQNCLQWWEEEQ-----------------DLDAGEKKDFSEEVAGKTYVKKKTSVVPQGKE
telata              EPSFPSKKRDSHSPCNHSSAPGIVMEPPLQRKKKPVTPMTTSTTQWHEES-----------------------GRGSDYILLGNQ---------------
Medaka              SAGALRSSAHRKPN-----------------------KPPTGSA--GNPE-----------------------SLSEPEPPAGPRPP-------------
tilapia             EPGLQRLRRPLGPA-----------------------QSSTSLLHPPGDE-----------------------QQHSLAHRAAPSA--------------
stickleback         EPGLQRRKRLGPPG-----------------------PAESCSVLLCPPD-----------------------PRNSSAPPAATQSP-------------
molly               PPGLQRLKRPG--------------------------PPESGNFLPGSPA--------------------------DPVCRATPSTG-------------
platyfish           PLGLQPLKRPG--------------------------PAESGDFLPGSPA--------------------------APVCRTAPSTG-------------
mouse               EPGFISKRRVPYHD-----------------------PQISKYLEWNGTV-----------------------RKKDTLVPPEPQAF-------------
human               EPSFISKRRVPYHD-----------------------PQISKSLEWNGAI-----------------------SESNVVASPEPEAP-------------
cat                 EPSFISKRRVPYHD-----------------------PQISKSLEWNGAI------------------------SENDMVSPKPEAA-------------
```

```
cattle              EPSFISKRRVPHHD-----------------------PQISKSLEWNAAT-----------------------SENDVVLSPETEAP-------------
python              EPNFISKRRVPYYD-----------------------TQISKSFEWNGDN----------------------GLEKEALSELEVSEP-------------
chick               EPNFISKRRVPYHN-----------------------PQISKSFEWTGDC-----------------------DSDDPVETEALKTA-------------
xenopus             EPRFPSKRRVPYYK-----------------------AQISKSFQWKEPR-----------------------DNGYNISESIMEEP-------------
shark               EPSFISKKRVPYYR-----------------------PQVSKSFQWDWNS-----------------------NSGQDLQEPKPKES-------------
gar                 EPSFTSKKRVPFYR-----------------------PLISRSFQWEQGD-----------------------GSERSQPAAAEERP-------------
zebrafish           EPQFISKRRVPFYP-----------------------SQVSSSFRWEGRD-----------------------HSQQQLEKSRSPAV-------------
tetra               -------------------------------------PQ-------------------------------------------------------------
cavefish            EPQFQSKRRVPFYP-----------------------PQVSSSLQWRDSA--------------------------------------------------
limpet              EPSIQQKKRVEGPTTSMSCDFFQR-------------PPPEEYIVLGGQDKFSTNNVKYVPKKRLVMDNVNRSEANSHFIQPQPDEPIRSMDFHLKK---
oyster              EPPLQRKKRCTGPP-----------------------TTLSTDFNQFPDA-----------------------ASDDFALLGRNDKFMENVRYKAKSHHT
aplysia             EPPLQHKKRLEGPPTSLSTNYFSV-------------PSKEMDYALLGQQ-----------------------EKFAPNVRYQPKPS-------------
Clustal Consensus

                             310       320       330       340       350       360       370       380       390       400
                    ....|....|....|....|....|....|....|....|....|....|....|....|....|....|....|....|....|....|....|....|
daphnia             ---------------------------------------------SAAAVGSSSG---------------------------------------------
silkworm            -----------------------------------------------IRPQGSAADRARSAQRNDADR--------------------------------
butterfly           --------------------------------------DIRPQ--QASAFERARS-AQRNDSER------------------------------------
bee                 ---------------------------------------------PTSTKQASAK-LQSAKSTEDENN-----------EGPTLEPP-------------
fly                 --------------------------------------TDGRR--DASAHRLARS-EERGGT--------------------------------------
mansoni             --------------------IQTDGAHKALEEEIRRIRTEIENRKRILRAREEAKENKKHVKIENEIK-----------QKPHTK---------------
viverrini           ---------------------QTEGPRKLLEEEIRRIRAELEQ--RKHRLKNVET-TSQPLPQNSQPH-----------ESGELKLKL------------
sinensis            -----------NQRHYKRESTQTESPHKVLEEEIRRIRAELEQ--RKHSLKNVET-TSQSLPQNNEPY-----------ESGELKLNL------------
anemone             ----------------------------------------------------------------------------------------------------
Trichoplax          ----------------------------------------------------------------------------------------------------
urchin              ---------------------SSLSKTFLKKVDMPAKTRPPID--KTLKTEKNKA-LAAQTRVISKPS----------QPPPPPKKVA------------
Saccoglossus        ----------------------------------------------------------------------------------------------------
ciona               KIMNGVGNKTYAKK-------TKDSSPGRDEIKRSLVERVDAK--LDATKRIKKV-AKQDKQVSENNQTNKKTIREFSKPQPRPEVQHSLTRKHSP----
telata              ---------------------GKFASNVQYNPKRAWKKKEPVK--ESSPRSKPAV-PSAAPDKATPTS-----------PPPAPRLIAPAPKVVVDTGAP
Medaka              ---------------------A----------------D----------PCPPKR-PGLEPSV----------------RPAAVGESD------------
tilapia             ---------------------------------------------DCQVLGATQ--PTGGCPPQSPIH--------------------------------
stickleback         ---------------------SAHGKKTALEP-------------NPPTPPGPRADPGLGTQGAGPAA-----------DPGSVEPGAAGKSA-------
molly               --------------------------------------RKDSPVSKPPNVSGSEPEPGFRPAADGKSV--------------------------------
platyfish           --------------------------------------RKDSPVSKSPNVSGSEPEPGSRPVADGTSV--------------------------------
mouse               ---------------------GTPKPQEAEQGE-----DANQE--AVLSLEASRV-PKRTRSHSADSR-----------AEGVSDTVE------------
human               ---------------------------ETPKSQEAEQKDVIQE--RVHSLEASRV-PKRTRSHSADSR-----------AEGASDV--------------
cat                 ---------------------------ETELQEAEQKEDVNQE--RVLALEASRV-PKRTRSHSTDSR-----------AEGASDIVE------------
cattle              ---------------------------RTPESQEAEQTDVNQE--RLLAVEASRG-PKRTRSHSADSR-----------SEGASDIVE------------
python              -----------------------------IELHRNGSKDVEMNQEPTETPDAPRV-PEKVHLNSSGSR-----------PESTVAL--------------
chick               ------------------------------ESLAEHSNDVNQE--NTETPEGPRL-PPKVRSHSSDSG-----------VETALALAG------------
xenopus             ---------------------------------VQLRLDLDRESLAEEKLHTPDA-PRLSEKANNYAV-----------SSKMVKDVG------------
shark               ---------------------QPVKSQTSVLSE-----GMNAE--DCKTPEAPRS-LRFGRSRSMDCR-----------LHSALEPPM------------
gar                 ---------------------------------------LSPE--RTETPPAPRV-PRLPRSPSEDLN-----------PQPFPVANG------------
zebrafish           ---------------------------------SPVLRATSAE--RQVTPLAPRDPPEG-----------------------------------------
tetra               ----------------------------------------------------------------------------------------------------
cavefish            ----------------------------------------------------------------GSLR--------------------------------
limpet              ---------------------DKEIQVETLKPAKSPPKVNTRQFTDPLTPLAPRE-PKYPTETHATQT-----------LNQEPQLQHKQKSHLNKNGLE
oyster              APAREASPPKQQRLQEKENRRKAPKPTPAPFVDTTKFRQQSKS--EEQQPSTPLT-PRKEKNESSSLY-----------KEIALREKEAALRQKFSKLDR
aplysia             ------------HSGQKIYKNKENFDNAPPPSKQLKMQKRIKPLFKERHPTVSRSPPRQAPTTSTILS-----------PPPAPRVPKESLDIKEALRDK
Clustal Consensus
```

```
                              410       420       430       440       450       460       470       480       490       500
                     ....|....|....|....|....|....|....|....|....|....|....|....|....|....|....|....|....|....|....|....|
daphnia              -LAAENRAGAPPGSFSQQQ---------------------------------------------------------QEQQQHNQKRVVVLAGETTANKRT
silkworm             -AGRRSKSEGPRQPRWTDTVE-------------------------------------------------------PKATTALGEVVVAKEPDMVS----
butterfly            -AGRRSKSEGPRQPRWTDTVE-------------------------------------------------------PKATTALGEVLAAKEPETVS----
bee                  -LPRRKKCPELAYKTHEFITAADGGGIDAVD---------------------------------------------SNVVADKVREVTTQSGLPPSQLSK
fly                  -PSRRSKSEGPPVVPNGRA---------------------------------------------------------YPIATEIDGTTRKQAGESNGLLKK
mansoni              -TPKPIESDYPMVTRAPDATRQHKSN--------------------------------------------------KMPLQEYSENWKLEPPKAAV-YTC
viverrini            -GDSPVVTGSHKTPQAPRAVRKKGTLE-------------------------------------------------YIVAPNIENKRRRKSYSPLAQPRL
sinensis             -KDSPVVPGSHKTPQAPRAVRKKGTLE-------------------------------------------------YIVAPNIENKRRRRSSSPFAQPRL
anemone              ----------------------------------------------------------------------------NHEDDKENRALEYKAGLAPAMPRT
Trichoplax           -------PDDTPQSLVQEVRQKAAI---------------------------------------------------KDEDKIGSKALQYKAGLRPTNPPR
urchin               -PPSPIRAAKGPAITKTGKHVPKAGGDASKTK--------------------------------------------GKAEQETDEALQYRAGIKAR-P-G
Saccoglossus         ----------------------------------------------------------------------------------------------------
ciona                -KRKSIQEQSGPVSPTKSIKSPKSSP--------------------------------------------------KKETKSKNIPLRYR---SLN-SRL
telata               KKTRPVKPTSPPAKKTKVVAIETKAPEEAKKLKESVNLSKMEAKKENDARRVYEVQLAKEEDLIRRQVEKEREIEQKKKALKGNYAMKYRSGVAPP-RSR
Medaka               -KPHPFRGHSQPQPSSPVPAAAANELQP------------------------------------------------PPPAMKADHVTRRRAGSKAP-GHG
tilapia              -QHPPEEIPDPPQSRKLQPDHDLLRNR-------------------------------------------------QLMENLIDYALRQKAGLRSG-GQR
stickleback          -KPRPSKLDSRPQPSQPLTSAP------------------------------------------------------DGRANEVQRALRWRVGLK---GQR
molly                -KLRPSEPGGPAEPE-------------------------------------------------------------QQPGNQVDKAWGQR----------
platyfish            -KLRPFEPGGPAEPE-------------------------------------------------------------QQPGNQVGKAWGQR----------
mouse                -KHQGVTRSHAPVSADVELRPSSKQPLS------------------------------------------------QSIDPRLDRHLRKKAGLAVV-PTN
human                -ENNEGVTNHTPVNENVELEHSTKVLS-------------------------------------------------ENVDNGLDRLLRKKAGLTVV-PSY
cat                  -NNEDVIEGHIPVNENEELEHSTKLLS-------------------------------------------------ENVDNGLNRVLRKKAGLTVV-PSH
cattle               -NKEDIKANDIPANENVELKHSTKPLS-------------------------------------------------ENVDDRLNRLLQKKAGLTVV-PSH
python               -GRSKKSVSQTPVHENKVLASTKKDL--------------------------------------------------GKTDHGLNRVLQRRAGMNIS-QSR
chick                -NSMKKTPPVAPPNQKEAFVSPKKEA--------------------------------------------------EKVNNGLHRVLQRKGGMNTP-HLS
xenopus              -STVKKTAIEAPEDNRETVAPSKKVNS-------------------------------------------------QAKANGVHRVLKKKAGLSTA-PLQ
shark                -AGDKPQVAEAQKSKKDNNTPPKKEGAD------------------------------------------------EGIANGVSVVLQKKSGLKTA-PRK
gar                  -KNLPSSDPDKHCPTRTAAAAERS----------------------------------------------------DAGLDGVHRVMQKKAGLKST-VRR
zebrafish            -TTAPSQPPQAEAAQTSTFAVQKQ----------------------------------------------------KQALNGINHALRMKAGLRSE-HQR
tetra                ----------------------------------------------------------------------------------LNYALRKKAGLRTA-RQR
cavefish             -QQEIARPGSPPASVHVTRTS-------------------------------------------------------------------------------
limpet               KSSSHHDTHHAHQQNQERQNIPKTHKTKMAARKNPEEIRYKAGLKDKNIQDVLENKKFPNKMNEFTQANIKKGILDSAPEATADYSLQYKAGVA---PLR
oyster               EGGRKTAPSALRQSKEQYSKTIDDNRME-------------------SAVKSWVKDKNVGSMNEFAQTNLDKGIADSAPEAPADYALRYKTGVSVPRPRR
aplysia              -DLKDVLGDQAPPPKEAGVKTRESDAQL--------------------------EKGRGNNMNEFVQTNMKKGVALSGPEAPAEYALKYKAGVAPPRPVK
Clustal Consensus

                              510       520       530       540       550       560       570       580       590       600
                     ....|....|....|....|....|....|....|....|....|....|....|....|....|....|....|....|....|....|....|
daphnia              ISTTGSSEYQLQFAW---------------------------------PPS---GV--KNISNTSSAFSN------------------------------
silkworm             ------TEYRNQFAWPKD--------------------DT--------TPR-------KSISMGALKKAA------------------------------
butterfly            ------TEYRNQFAWPKD--------------------EV--------VPR-------KSISMGALKKAA------------------------------
bee                  AISRISTEYRLQFAWPRR------PQLTNGETVAPGVTAAGLPAGTTGPPR-------KSLSMGALKQGI------------------------------
fly                  TINKLSTEYRLQFVWPTV-----RRIKGGGEATSRAAAGD--------YPR-------KSISLGALRSG-------------------------------
mansoni              EKSRL-SEYQSAFKAPQF-----NYKLEPTKSTRPFTCKL--------DKF---QS--CKISYDTEYRHE------------------------------
viverrini            ------TEYQRAYKAPELTPIPQRPRTAQSE--FPTTFKA--------SSH---HG--QIDIFETEYARE------------------------------
sinensis             ------TEYQRAYKAPEITSVPQRPKTAQSE--FQTAFKA--------RSY---HG--QIDSFETEYARE------------------------------
anemone              KKQRYVSEYQKQFYWKKP-VGREAPILSAEKVVFSSQDNL--------PPA---KPVKISMPTKSEYQLQ------------------------------
Trichoplax           LAYGK-SEYQRQYDWKKL-PERASPIIAADNTIFKSSTDI--------APVKVDMG--PRPNVKSEYQRQ------------------------------
```

```
urchin              GPRYS-SEYQRQFAWKQP--SKESPLMAAEQMVHTSNANL--------APY---VP--DKIPRHTEYQRQ------------------------------
Saccoglossus        -------------------------------MIYNSN-AI--------QTY---MP--KNVPSKSEYDAQ------------------------------
ciona               MSAHQ-TEYQQMFRQPAR-FVPSSPLISALDVVHQSSAAI--------PPHVINKS--PKMRLTTEYSSK------------------------------
telata              GPKRL-SEYQLQFKWMKG--VANSPLLAADQVVHKSNTQI--------GPY---KT--ETANKKSEYATQ------------------------------
Medaka              SGRQ------------KP-LMAASPLLTAEQVLYAGSRGI--------TPY---RM--KPVSMETEYRRN------------------------------
tilapia             SGGKH-SEYHRQFGWKKPTAAAASPILTAEQVLHSSSRSV--------PPF---KI--NPVSMETEYQRS------------------------------
stickleback         SGIQR-TEYHRQFSWKKP-ATAASPILAAEQLLYSGSRCV--------PPF---KK--HPIPLETEYRRS------------------------------
molly               ------SEYHRQFGWKKP-VSAASPLLTAEQVLHSTSRSI--------PPF---KN--IPVPMETEYRRS------------------------------
platyfish           ------SEYHRQFGWKKP-VTAASPLLTAEQVLHSSSRSI--------PPF---KK--NPVPMETEYRRS------------------------------
mouse               NALRN-SEYQRQFVWKTS--KESAPVFASNQVFRNKSQII--------PQF---QG--NTFTHETEYKRN------------------------------
human               NALRN-SEYQRQFVWKTS--KETAPAFAANQVFHNKSQFV--------PPF---KG--NSVIHETEYKRN------------------------------
cat                 DILRN-SEYQRQFVWKTP--KEAAPVFTANQVFHNKSKFV--------PQF---KG--NSIIHETEYRRN------------------------------
cattle              NVLRN-SEYQRQFVWKTP--KQPAPAFAANQVFHNKSKFI--------PQF---KG--NSFIHETEYKRN------------------------------
python              SFPRS-SEYQRQFVWKTP--QKLSPVLAADQFIHSSSQSI--------PPF---KS--PVIIPETEYERN------------------------------
chick               TFPRN-SEYQSQFVWKSP--HEKSPILAAEEALYNVSKPV--------PPL---KS--PAITSETENVRN------------------------------
xenopus             HPLKM-SEYRRQFERKTP--TENSPMLAAEQVIYNKNKSI--------PPF---NV--DALKTETEYKSR------------------------------
shark               NLARS-SEYQRQFSWKTQ--MENSPLLVADQVIHNRNQEI--------PPF---KS--GKVCRETEYSSQ------------------------------
gar                 SAAQN-SEYQRQFSWKTA--PVSSPLLAAEQVIYNRNRDV--------PPF---KS--DAVVLESEYKKN------------------------------
zebrafish           NGLN--SEYQRQFMWKTP--VAESPLLAAHQMLYSNNRAI--------PPF---KT--NPVIMESEYKRS------------------------------
tetra               NGRQS-SEYQRQFLWKTP--ANESPLLAAQQMLYTSNRSI--------PPF---KF--NPVEIETEYKRN------------------------------
cavefish            ----------------------------------------------------------SPEKVETPLAPRTSKPQRAKKTSQHVESVSCSQVRSPSADQQ
limpet              KGRKW-SEYQKEFDWKNR--VESSPLIAADQVVHKSNTVL--------VAP---KQ--IPKPRASEYKSQ------------------------------
oyster              HKF---SEYQKSFSWKDG--AKASPVLAAEQVVYNSNPAL--------VP----PQ--KSFVKDSEYAAQ------------------------------
aplysia             KF----SEYQRQFQWKDG--MKASPLLAAEEIVYKSNAEL--------GPY---KT--DAIPKLSEYRQQ------------------------------
Clustal Consensus                                                                   :

                             610       620       630       640       650       660       670       680       690       700
                    ....|....|....|....|....|....|....|....|....|....|....|....|....|....|....|....|....|....|....|....|
daphnia             ------------SRRMP--ESLDSLLNH---SQDGEREAAGLLSSRINNGTGTTTRSQEMVY-----------------------HAAEELLLNHQQQQQ
silkworm            ------------AAEGS--VEVEPLMNH----VDGDHDEGHKRYIEDYLWNGQKPGVQRKST----------------------------FRNALSA-LI
butterfly           ------------VAEGL--VEAEPLMNH----VDGDHEDGPKRYIEDYLWNGQKPGVQRKST----------------------------FRSALSA-LI
bee                 -------------------APTGPAPVHKKRPGDVDHKRDGMQASE-LEPLVGGTGTDTIDG------------------------VVPEGDEREEDMSD
fly                 ---------------GQ--GHCHSHTQN-----QNQSQSQGLTQAQNGHTHHTMMGGGAGLP---------------TVHKKRTTNQKEATLHELEP-LV
mansoni             ------------YKPFK--YVPIDQLKS----------SEVIENKDVHIIPLKRPSSAYGIR-----------------EITMNNINDTEKRRIRALTPP
viverrini           ------------YKPFR--YVPIDKLNA----------TTFIRDPNVNIVPIPRPKSAYTLV----------------RSQQIKDTENGYPRSRQETSSQ
sinensis            ------------YMPFR--YVPIDKLNA----------TTFIRDPNVNIVPIPRPKSAYTLV----------------RAQQPEDKANDYPRIRPET-SH
anemone             ------------FVKYP--LVTTLR--------DDECAGSQGKEKEAGANKKAELQPPAGVA----------------------------SSPDKQQTPL
Trichoplax          ------------YQDWR--QSMQQSTKP-----AAQDQSSRLARPSRSIDERSESSLDTTKSILDGDYYNKKEKSEDIKASGYKDKISDYMERLEQS-EN
urchin              ------------FKQQN---HGGPLVQD-----QLMQNAQQEIKAKYRVKTKSKRGKKLTPD-------------KQQPAGVLSVISHDDPRLVKSS-QN
Saccoglossus        ------------FQAWN--ESRPSQVEM------LKEHAKRDAELEVKQKKKSSKGKRRKSG-------SKKEVVTPDQMPAAGVPTFHLPEQLKNA-QN
ciona               ------------YQNRS--PL-----------------------SKSMTLPTSPASPSLKMK---------------------------------VK-NL
telata              ------------FKQYQ--PVQSAEPAR-----KPAKVKRSRSLEAVVSPDHLPPAGLNRVT-------------------------ADFDPRLVQESLN
Medaka              ------------FRGLI--PPTGPRLRN-----YLDHELE----PLFHSYEVNRRTREEEPA-------------------------SKLRLKHDSHPEE
tilapia             ------------FQGLA--PPAGPRLRK-----HLEHQRV----PLFQTYKETRRERRDEEE--------------TTRRRKEESQKKPRLQRDALQ-RD
stickleback         ------------FRGQV--PSPGPRLRE-----HLEHHQRA---PLFHVHTTNKKKREESVK---------------------------KTRPRQDN-VT
molly               ------------FQGLV--PPSGPRLRR-----NLGH-------PVFHTYRTHRRKSLDAQS-------------------------NELPVKQETGSQL
platyfish           ------------FQGLA--PPSSLRLRR-----NLGH-------PVFHTYRTHRRKSLDGQN--------------------NKLPVKQETTQGDGGSRL
mouse               ------------FKGLT--PVKEPKSRE-----YLKGNSS----LEMLTPVKKADEPLDLEV--------------------------DMASEDSDQ-SV
human               ------------FKGLS--PVKEPKLRN-----DLRENRN----LETVSPERKSNKIDDRLK-----------------------LEAEMELKDLHQ-PK
cat                 ------------FKGLS--PVKEPKLRN-----DLKENGN----FETVSPEKKCNKTDDPLK-----------------------LEAEMESKESNQ-PK
cattle              ------------FKGLS--PVKEPKLRS-----HLKENGN----FETISPEKKCNKTDDPLK-----------------------SEAEMESKDSNQ-PK
```

```
python              ------------FKPSS--PIKELKQTS-----FLEESEC----PRSESVEVSSGEKNEKNK--------------TTKAIEDSSNQIKLETKQKEE-KL
chick               ------------LKGSP--AAKGPQEKC-------SEEKEFLVHEQTKREELIQKPAEDASK---------------------QEKSEQKHPKRKNKQHI
xenopus             ------------FKGSP--PAKGPVLRK-----DWEEKHVPKYEPEDPSIKRKEKRIQINEA-----------ICSPPKNDKSETEAKQRIHARKHKEKV
shark               ------------FQGTP--LPKGPRFRK-----DFEEKEALFFEPENDSPPKQTPSSSKQVG----------------------ARIKEQESEQLLKQQN
gar                 ------------FKGLV--LPKGPRLRR-----DVEEREAVLYEPENISPDKTPKSKKNKKG----------GSTKHNNTSSVPHPRRERERQVSRK-QN
zebrafish           ------------FKGSP--LPRPPRLRR-----DVEQYEVPEFLTESKTPEKSKRKKKKKER--------------PHSRKSSPEQEVAYLQQQEVK-SP
tetra               ------------FKGSP--PHRPPRLRR-----DVELNEL----PENISSDKQEREKKREQR--------------LSRKPRSREDSHPEPQPVQQE-VI
cavefish            EQEHPSNGIPFCYESNN--QIKSNQIYLYSAFYNWCWHKAALQKLNKGIESIPEPSCFEKLG---------VRNNYDKMKPRSREDSHPEPQPVQQE-VI
limpet              ------------FKAWK--VKPVPVGRK-----DVNEE------PEIKKAKIKRNKSMGAID--------------------------RKVQVDKENIEA
oyster              ------------FKQYNAMPVSSDDLKA---IPRAEKPRSKVKRSKSTGALRVDKRPAAGSG--------------PLITPDNKRQTIESELQGEPQ-DA
aplysia             ------------FQKWKA-PDFAADVNL-----NVSEKMATAASQQKQEKKRTVAKRSKSAG------------AVLHQEPEEVDDEMGAEGADSEE-QL
Clustal Consensus

                           710       720       730       740       750       760       770       780       790       800
                    ....|....|....|....|....|....|....|....|....|....|....|....|....|....|....|....|....|....|....|....|
daphnia             QNELCDDQPRRRRYKTEYKKKFRPFSLYQYVD--GKFQKSSSDKIPTSSST-----------------------VDGPARQQQQPGDKMDKDTWYGEVLE
silkworm            SNGEDRSKDNRKRYKSEYKKKFRPFSQYVYEASKGKFTKCRGKGKSNEEASERMLGENQGSGEGGQGGGVEDS-ASTLRAAGLQPLGLAQGDSWYREVLD
butterfly           YNGEDRSKDNRKRYKSEYKKKFRPFSQYVYEASKGTFTKCRGKGKSIEEASERLVGDTEGAGAGAGDGAAVEDTASTLRAAGLQPLGLAQGDSWYREVLD
bee                 LKITFRGKKSGRTVRIS------------DDK--G---------------------------------------LREKPQEKPFPT---------AEVIE
fly                 SDTDDRKTHEKQVTIMERKTTSRPFSQAIDQERLNHFITKKENFGFADAAVAAAALKDEVDNRQAGESG-----QVVVMNGSAPPHSKPNLDLWFKEMVE
mansoni             SQIHTKPIQHRKKYTTEYNAKYID-----YSD------------------------------------------VLDVPQNQMKSSNKSISHDWYQETVV
viverrini           PDLSKKTRSHESRYTSEYGAKYHN-----YFG------------------------------------------LQPNSNGSTE---------WMRVLEG
sinensis            PDLAKKTKSHESRYTSEYGAKYHS-----YFG------------------------------------------LQPNSNGSTE---------WMRVLEG
anemone             PLAPEVPYKPM-KFQSEYHTNFQPPSMFDYID--GAWVGADP--------------------------------PHLYPLEKPSDDVHPVTLSWFKQVTE
Trichoplax          PDRPFFPHRRRNYLLTYFYDLYIQDDDNSPVI---------------------------------------------SPDGTPS---------WYKQILE
urchin              PQRPFFPHATVRKWRSEYKSNFKTPQVFSYEN--GVWKGADPPHVQPRDESSD---------------------RGGKPEQLTRSKSVGDLPNWFSEVLE
Saccoglossus        PQKPFFPHAAIKKWKSEYRSNYHDPRVFSYMD--GVWRGADPPHIEPRDESSKQEKIGVAPDWFGEVRGT----PKQFSNYSDKKKDSVHDAKWFKEVVE
ciona               PFSPVQHHRSPKKVISEYSTQF--PDREFPLE--P--------------------------------------------------------------LSN
telata              PNHFSFPHGKFKSSNSEYHRNFRPPKDYVYQD--GAWGGVVP--------------------------------PRVEPVMEPEAEEGAPTNSWYSEVME
Medaka              EDTPPLLGHRGRRMLTEYQSSFRSPLHRKPEG--N------------------------------------------TNGAALQ-------------VTE
tilapia             MPPPPRQVRRGYRMLTEYESSFRSPLCRIPEG--GG----------------------------------------AMDSTAQQ-------------VKE
stickleback         PTQVQRGPRI-------------------PEG--GG-----------------------------------------ATEGDAQ-------------VRE
molly               PLTTAPPPAQVRRRHRN------------PKG--GG----------------------------------------TTDDLTEQ-------------VKE
platyfish           PLTTALPPAQVRRRHRN------------LKG--GG----------------------------------------ATDDFTEQ-------------VKE
mouse               KKPASWRHQRLGKVNSEYRAKFLSPAQYFYKA--GAW-------------------------------------TRVKENLSNQ-------------VKE
human               RKLTPWKHQRLGKVNSEYRAKFLSPAQYLYKA--GAW-------------------------------------THVKGNMPNQ-------------VKE
cat                 KKLTPWRHQRLGKVNSEYRAKFLSPAQYLYKA--GAW-------------------------------------TRVKENIPDQ-------------VKE
cattle              KKLTPWRHQRLGKVNSEYRAKFLSPAQYLYKA--GAW-------------------------------------TRVKENIPNQ-------------VKE
python              KQKPLCSHRSLKKMNTEYRSKFLSPNQYVYKQ--GAW-------------------------------------VHVRKNIPYQGSQNTLNTMWYIEVKE
chick               SQKPLSLHTHRGKMNTEYRSKFLSPAQYFYKD--GAW-------------------------------------SRIRSKVPNQASQNPLNSMWYMEVRE
xenopus             IQQLKHSSKSCRRIKSEYTSNFLSPSEYLYKD--GAW-------------------------------------VRAKKQMHEQ-------------INE
shark               PHKPPFPHKGHGKIKSEYRTNYLSPDQYQYRE--GAW-------------------------------------KAKRSFVAEEGTESKLSFPWYAEIKE
gar                 PKRPFFPHRGCGKQKSEYSANFRSPLLYRYKD--GAW--------------------------------------IKSPCVGEEGSQRTLNHMWYNEVKE
zebrafish           HNLKDPSPKVMRKGKTEYRSNFHSPLQYSYKD--GAW-------------------------------------LKIKSAKEE--------------VKE
tetra               SSQKDSSPKVLRKVKTEYTSNFRSPLQYRYKD--GAW-------------------------------------IRSRVSGEEG-------HEWYHEVQD
cavefish            SSQKDSSPKVLRKVKTEYTSNFRSPLQYRYKD--GAW-------------------------------------IRSRVSGEEH---GQMGHEWYHEVQD
limpet              GEVIKSTHGKLRKRESEYDANFKAPQEFQYMN--GAWHGANPPQLYTSIKPVRQVCCSVKLWSKFAVRPKPVKKSKEKKALVEAEDIQPEQTNWYTEVLE
oyster              PSSPPIHQGNLKRPRSEYTSNFRSPTKFQYD---GAWHGADPPHLQPTREPEK---------------------EKPVKKSGPVEDSAPALSNWFAEVIE
aplysia             KTKLLKSHGKIKRLGSEYKANFKSPLRYSYAE--GAWRGAAPPQMMPQTSSAENTDAN----------------NDKENNNNNNASEEAPLSNWFAEVLE
Clustal Consensus
```

```
                                810       820       830       840       850       860       870       880       890       900
                       ....|....|....|....|....|....|....|....|....|....|....|....|....|....|....|....|....|....|....|....|
daphnia                LRKKAGEYKYRGL-GGELMSDHMADLYAKQFDVWEQVSRRSSLS---------------ALALAATSPRPGSE---------------------------
silkworm               LRKRAGEYKYRGW-GTELAPEHLTQLYNKQIELWYQVSRRSSLS--ALSLASTNHKVLPRDEKDGKESKNQSP---------------------------
butterfly              LRKRAGEYKYRGW-GTELAPEHITQLYNKQIELWYQVSRRSSLS--ALSLASTNHKALPRDEKDGKESRNLSP---------------------------
bee                    LRRLADEYKHRDW-GCGLAAE-------DEASLWKRVSSNHALN------ALSLARSVTKEEKEKENTRKVAPVTAAMTMPPAGRPSSVQARPIHGILQD
fly                    LRKKAGEYKCRGW-GIEIDPE----LYKKQKDLWDQVSKRSSLS--ALSLASSVHRPITKEEKEQENNKKSTPLQKAQKPRVPGQAFLIDNKDEISALPA
mansoni                LRKKADAYRKRDR-ESHFSREHLAQLESDYVDHWDPPSDDNYME--------NLEKYREVLQMPRAGKPSTDL---------------------------
viverrini              IRNQADLYRKRDL-ESHFTRDHLAQLASAFVDYWDQPSSDRYAR-----------NREKSLTTLAQPPAGKPS---------------------------
sinensis               IRNQADSYRKRDL-ESHFTRDHLAQLASAFVGYWDQPSSDRYAR-----------NREKFLKILAQPPAGKPS---------------------------
anemone                LRNKAQSYKTRAR-GDSQFLGILKSLEEQQSITLEKANKRTPSA--QHRLRDDEANRVPDMRIECLEERQEKA---------------------------
Trichoplax             LREKVQLFRERSQQGTFFTRKNLLVMEMAQEDLWEPASDTSRSG--------------------------------------------------------
urchin                 LRERAKEYQKRSR-GTHFTRQHLGQLLANQAKLWEESSDQLSTS--------------SSISTQTTGSEKKDE---------------------------
Saccoglossus           LRKKAQEYQKRAR-GTHFSREHLAQLLAQQVKMWEMESVSSTST---------LTPSERTVSTARPAAGKTYT---------------------------
ciona                  IHQEANQNKQNRD-ANSFDPDHATQLHSPKNKFWEIMSATESNV-------DTVSNSTSSVKKVVESLKSKDV---------------------------
telata                 LRKKAAEYRLRAR-GTHFSRSHLAQLYAQNAELWDSYSVVSALS--------------------------------------------------------
Medaka                 LRQRALMYRRRAW-GANFSRDHLGQLLSHQNALWEPSSTTDSMA----------DASPLAAGPSPDPGSHSGS---------------------------
tilapia                LRQKALLYRHRAW-GTNFSRDHLSQLVSEHNALWELTDTTVSAN--------DPASPRLPPNPSPDRDSHSTS---------------------------
stickleback            LRLKASSYRRRAW-GANFDRDHLSQLLSEHNALWEPTDTT------------DGPTPRPTFDLHADPDSCGTS---------------------------
molly                  RRQQAQAYRRRAW-GTNFSRDHLSQLLSEHNALWEPADSHSDPS-----------TPLPNRDLSPEPDSRRAS---------------------------
platyfish              RRQQAQAYRRRAW-GTNFSRDHLSQLLSEHNALWEPTDSHSDPF-----------TPPPNRDLSPEPDSRSAS---------------------------
mouse                  LREKAESYRKRVQ-GTHFSRDHLNQIMSDSNCCWDVSSVTSSEG--------TVSSNIRALDLAGDLTNHRTP---------------------------
human                  LREKAEFYRKRVQ-GTHFSRDHLNQILSDSNCCWDVSSTTSSEG--------TVSSNIRALDLAGDPTSHKTL---------------------------
cat                    LREKAEFYRKRVQ-GTHFSRDHLNQLLSDNNCCWDVSSTTSSEG--------TISSNIRALDLAGDPTSHKTL---------------------------
cattle                 LREKAEFYRKRVQ-GTHFSRDHLNQILSENNRCWDVSSTTSSEG--------TISSNLRALDLAGDPTSHKTL---------------------------
python                 LREKAEAYRQRIQ-GTHFSRDHLNQILSDNNRFWDLSSESSVEE--------TMSNNVRALDLARVPENKQSL---------------------------
chick                  LRARAKAYRQRIE-GTHFSRYHLNQLLSDNNSLWDVSSNSSSEE--------GISNDIRALDLAGVSEKETAP---------------------------
xenopus                LREKADYYRRREQ-GTHFSRNHLNQILSVNNKFWDVSSNSSSEE--------QVSHNIKALDLAGFQAHSKKE---------------------------
shark                  LREKAEVYKRRAL-GTHFSRDHLSQILSPENKLWELSSTSTSEE--------SVCDSVRALDLERARETPKIP---------------------------
gar                    LRERAEAYKKRAW-GTHFSREHLSQILSEQNRLWEVNSSSTVTE-------DTVSDTVNALDLARARDVKRSP---------------------------
zebrafish              LRERAEAYKKRAW-GTHFSRQHLNQILSDQNWMWEPSSGTSSSS-IESEACRSTSHIIEALDLARAGSVRESS---------------------------
tetra                  LRAKADAYRKRAW-GTHFSRQHLSQVISEQNCLWEASSASSSSTLTEQDSHSHSSPTIEALDLARVDSVRGS----------------------------
cavefish               LRAKADAYRKRAW-GTHFSRQHLSQVISEQNCLWEASSASSSSTLTEQDSHSHSSPTIEALDLARNRVLVYQV---------------------------
limpet                 LRQKADEYRKRGQ-GSHFSRAHLAQLLARQTELWDCETCSTTEA-----------STISALSLETGSSPAGLA---------------------------
oyster                 LRRKAAEYKKRAQ-GTHFSREHLVQLIAQQNQAWDNLSTARSGS-----------STLSALSLESGASAKLRA---------------------------
aplysia                LRRRAQEYRKRAQ-GTHFSREHLVQLMAKQNECWDLHSDGSSTV--------------RALNLETSGAHLRGK---------------------------
Clustal Consensus       :  .   : .

                                910       920       930       940       950       960       970       980       990      1000
                       ....|....|....|....|....|....|....|....|....|....|....|....|....|....|....|....|....|....|....|....|
daphnia                -------------------------------------------KVDMTSAIN----------SKRGSPAKTAPQPNQSARPGHAQPLV----------PT
silkworm               ----------KKFRS-----------------------FRSAPHQSIHAKLQETK-------ALERSPHKTSPQKQRKKLQGHSFDE-----------GA
butterfly              ----------KKFRS-----------------------FRSAPHQSIHAKLQETK-------ALERSPHKTSPQKQRKKLQGHSFDE-----------GA
bee                    DSKADTERAIARARKDFLIRHHLDRTTGVGDGALLPSPTREKLEPVIPRRREETKEHREEIQPKTKSSPKNSPRTGRSQSLGPTVTERRSPKRQPPRAPS
fly                    RFSNIRHHLERTTGPDVEEGALLPSPTREKLMPAITKRESESQRGSPKKTALSRHGSPQKGSPQKGSPKKVLKTRSQSAGPGVAENESPKRQIRS---AS
mansoni                -----------------------------------------------------------------------------IFNPDEAVLPA----------IQ
viverrini              ----------------------------------------------------------------------ELCSEENEECEAHSTI------------SA
sinensis               ----------------------------------------------------------------------ELCSEETEEREAHTTT------------SA
anemone                -------------------------------------------QPVSDHMIRKAE-------TGNQEKRNEFSGKNNNNEHGIHLVPTDDRASIDQGSGH
Trichoplax             --------------------------------------------------------------RENRTTNERGSYSTTSKKVSQVQQLP----------PS
urchin                 -------------------------------------------------------------------PKEGARPSASAPDLGHE--------------PT
```

```
Saccoglossus        -------------------------------------------KEVAAQQQAKLA-------REEERRKLTPSPSPSPHDSERTPQPA----------SV
ciona               -------------------------------------------DSCSVESIKGSD-------DDDCDVIRDENSVSCSSRQSLKGEL-----------PV
telata              -----------------------------------------------------------------------LETGSKSSRGGGDDSDR----------PS
Medaka              -----------------------------------------------------------------CVDALDLASVSSLSRSAASELKTR---------TQ
tilapia             -------------------------------------------GVEPLDLASHSS-------KRSSIAGSETQQMSKDTRIKKQAIPGP---------PA
stickleback         --------------------------------------------------------------PAEALSPARSSRRSSVTSSTDRNTPT----------PP
molly               -------------------------------------------CVEALDL------------ASMSSRRSSVSGGKTQRSAGRETPPGP---------GA
platyfish           -------------------------------------------CIEALDL------------ASMSSRRSSVSGGKTQRNAGRETPPGP---------GA
mouse               -------------------------------------------QKHPPTKL-----------EERKVASGEQPLKNSTRRLEMP-EPAA---------LV
human               -------------------------------------------QKCPSTEP-----------EEKGNIVEEQPQKNTTEKLGVS-APTI---------PV
cat                 -------------------------------------------QKCPPTTL-----------EEKKPALEEQPQKTTTEKLGVSDAPTL---------PV
cattle              -------------------------------------------QKCPSTQL-----------EEKRTVLEEQPQKTTTEKLGVSNAPI----------PV
python              -------------------------------------------------------------------AQPEQFQPNNSGNLGVSDAATV---------PV
chick               -------------------------------------------RPKMLQ----------------QPGSREQSHQDDTEKKGLSDAPTV---------PV
xenopus             -----------------------------------------------------------------NVHKSSIEENEDTGTLGISDVPTV---------PV
shark               -------------------------------------------RPKQQRSHSESA-------VENAAPSQNPPKFDDTGKNGISNVPTF---------PV
gar                 -------------------------------------------SPVSPGSL--------------SPVGNAKSRTEETEQAGLHSRPTL---------PV
zebrafish           --------------------------------------------------------------SPGHSASVVVSRRSSSGEVGLPEEPTL---------PV
tetra               ---------------------------------------------CSPG----------------ASVSLSTSRRSSFEGVGLSDGPTL---------PL
cavefish            -------------------------------------------IVCHPQTYCKM--------QVLSSLKQHVNMRSSFEGVGLSDGPTL---------PL
limpet              -------------------------------------------ERPPQ-----------------RPPRLELNEKNLERKSRIQDRKLKKFSDLD---ST
oyster              -------------------------------------------QKIELSEKPLLK-------IENIDTNNETLDNELCKGVRKSNDKMQQLKA-D---PV
aplysia             ---------------------------------------------------------------EKNLAQTAPADLVDALSHEATEGDS----------PL
Clustal Consensus

                            1010      1020      1030      1040      1050      1060      1070      1080      1090      1100
                    ....|....|....|....|....|....|....|....|....|....|....|....|....|....|....|....|....|....|....|....|
daphnia             S-----RNKDN-----------NIRNKGSDSTERARRDMSQHRSISSEGSKSRSKSAKPAKSPAQPTSGTGTGNVTLAIRPSLVSRPPARSASLGPERRM
silkworm            ALDGV-KTGEN----------------------------------------------SAFKSPRRR----------------------------------
butterfly           VQDGA-KSEGS----------------FRSPRRRPRS--------------------ADPAPAPPR----------------------------------
bee                 VTKDA-KEKEK-----------------ESKEKE-----------------------KEQREKGGE----------------------------------
fly                 QAPSS-RKKTPTTGSGSERKARPSTLSTTFQSRI-----------------------KSSSLPPPN----------------------------------
mansoni             RRRAM-LGSHN----------------------------------------------VAEIMDQDD----------------------------------
viverrini           RRRAM-MDSHH----------------------------------------------VAELLHEYQ----------------------------------
sinensis            RRRAM-MDSHH----------------------------------------------VAELLDEHQ----------------------------------
anemone             RKREA-WAEPG--------------PIYPRAAIPPYG--------------------VSESLPTSR----------------------------------
Trichoplax          QPKEA-WTVEA----------------------------------------------NENETEESN----------------------------------
urchin              RRKLA-WHANN----------------------------------------------NKAEEEPNP----------------------------------
Saccoglossus        KRKLA-WPVDE----------------------------------------------KPRQEEVSS----------------------------------
ciona               ARKLA-WGESD----------------------------------------------SQSNDVDAV----------------------------------
telata              VVAEA-WKEKD----------------------------------------------SKASNSTAR----------------------------------
Medaka              RRTHAETHTTA----------------------------------------------EEEQRPDG-----------------------------------
tilapia             ERRTA-WGEEE----------------------------------------------KEDEEEEEK--------------------REEEEETTDE----
stickleback         GQAA--RGGGG----------------------------------------------EYERSEE------------------------------------
molly               ERHSA-WAEEE----------------------------------------------EETEEEE------------------------------------
platyfish           ERHAA-WAEEE----------------------------------------------EETEEEE------------------------------------
mouse               RRKLA-WDAEE--------------STKEDTQEEPRA--------------------EEDGREERG----------------------------------
human               RRRLA-WDTEN---------------TSEDVQKQPGE--------------------KEEEDDNEE----------------------------------
cat                 RRRLA-WDAEN---------------TDEGVQKQPRE--------------------EEEEETEKD----------------------------------
cattle              RRRLA-WDAED--------------TT-EDVQKQPSG------------------E-EEKEEEEKE--------------------EGDK----Q-----
python              KRRLV-WDESG--------------NDEQQENQSPAL--------------------GECKEEEKK----------------------------------
```

```
chick              KRRLV-WGEQE--------------GTAEKDSQQ--L------------------T-EEEEKENEQ--------------------AAVVTQNLE-----
xenopus            RRRLA-WDENN---------------NSELAQERAALTVED----------------KNVETTDAD----------------------------------
shark              RQRLA-WNDEE--------------GETAPGRDLPES--------------------DQEESDGVE----------------------------------
gar                QRRLA-WEQED------------RGPESKGEEQGEHT--------------------GDLGRQEEE----------------------------------
zebrafish          QRKLA-WDEEE-----------QLGEREEIVQD------------------------RLTDKEGKT----------------------------------
tetra              QRKQA-WDEEERPYVNGVSEIKPLREEPPVQQA------------------------EESDEEEDT----------------------------------
cavefish           QRKQA-WDEEERPYVNGVSEIKPLREEPPVQQA------------------------EESDEEEDT----------------------------------
limpet             KATIA-ETPAP---------------SVNGGNERRTY--------------------KPRYTQDSR----------------------------------
oyster             VMETK-FNNKLRL----------ETKKENTNHRNVKTKSKKVEMANKIEDDVSLMAHEGSDEEQGKPDFVPEKHMDLTTKQRYKKSQSRKKAWQEMDESR
aplysia            MSDVE-EAEPQ---------------RKEGKKKKGR---------------------RGGKSKGGR--------------------KSRKMAWLE-----
Clustal Consensus

                            1110      1120      1130      1140      1150      1160      1170      1180      1190      1200
                   ....|....|....|....|....|....|....|....|....|....|....|....|....|....|....|....|....|....|....|....|
daphnia            PKSVSSSTTGGNVGSSATKASPVKARPAHNEDGIKDPVKVTMKNHLGDEGKENVIINEPASSSLPVIQQEPLPALIKALPEPTRVKSPEQLVVRSPDPVN
silkworm           -----------------------------PRSADPAPAHLKHAPNGHDARP-----IKPSDDEIP----SH-----------------------------
butterfly          ---------------------------AATDAERPHKPTGLPLSRGSSSRV----RSTSRGGRSP---SAP-----------------------------
bee                --------------PERHPRPTGVSVTGPGHVRWSIREPSTVSSTGSSTSVPPLPPPPSGPGPTPFYRRSP-----------------------------
fly                -------------GRVASAPP------ATAAAASAAKSALSANQAHANQSQSSHAKSGSISGRIS----KP-----------------------------
mansoni            ----------------------------------CSDTDYDQNIEYNN-------------DHLS----TQ-----------------------------
viverrini          ----------------------------------VYDPKSYSSEVKPESTH-------SKGEKNN----QR-----------------------------
sinensis           --------------------------------------VYDPESYSSEVKL----------GKWF----ST-----------------------------
anemone            ---------------------------SASSIGSDSMSDVTLTDDLMNERP---RSTPDDKMRKP----TT-----------------------------
Trichoplax         ----------------------------------KADEDYDDEETLSERPG---------NGNIE-----------------------------------
urchin             -------------NQNIAHLE------RRSSISSVTTPESMPSMSSRSSEI----ESIEENGRLT----TP-----------------------------
Saccoglossus       ----------------------------IGTIPTPESLQSSSRDTLTPNDD-------DEEGRIP----TP-----------------------------
ciona              --------------AHD-----------------EEDSDVASTVTLTESNT--------NEGRLP----TP-----------------------------
telata             -----------------------------------SNTVSVLNSEASESST----FAASEAGRLP----TP-----------------------------
Medaka             ----------------------------------------------------------------------------------------------------
tilapia            --------------SLLIKNE------KKTDEEIILKSQIEMSISAEENNQ----SSLAHSQKLK----TP-----------------------------
stickleback        -------------------------------------------------------------GRLP----TP-----------------------------
molly              -------------------------------------------------------------GRLP----TP-----------------------------
platyfish          -------------------------------------------------------------GRLP----TP-----------------------------
mouse              --------------QGKQTCA------VELEKPDTQTPKADRLTEGSETSS----VSSGKGGRLP----TP-----------------------------
human              --------------EGDRKTGKQAFMGEQEKLDVHEKSKADKMKEGSDSSV-----SSEKGGRLP----TP-----------------------------
cat                --------------RQVHNGE-------LEKVEVPEKSKAEKIKEGSDSSS----VSSGKGGRLP----TP-----------------------------
cattle             --------------VNVEELE------KLEV---QEKSKADKIKEESESSS----MSSGKGGRLP----TP-----------------------------
python             --------------QDIEEVKQVEKNDKDANINNQQKENISSSEDMVDSSE-----SLRTFGRLP----TP-----------------------------
chick              --------------KNNEGIN------EDNRIEGENTCLLNSPAAVSESSS----VSSEPGGRLP----TP-----------------------------
xenopus            --------------VVEDAEEMHAISEEVKRPDRLHSTTTNPSVDGLECIS----LPSEEGGRLP----TP-----------------------------
shark              --------------KELGNAEEVGAVSKRKEDPIDEANLSTANLDSNETSE----LGSEVGGRLP----TP-----------------------------
gar                --------------QADGNKKGDDAQAGQQQKEVKATEQRSSSRSSSDVTAGSSETSSDGGGRLP----TP-----------------------------
zebrafish          --------------RNENGME------RNERLNSLESESLSSAEEGSEASV--------NGGRLP----TP-----------------------------
tetra              ---------------NEHLED------KGEKFRVPVNEHLSPA-AGTRCSV--------EEGRLP----TP-----------------------------
cavefish           ---------------NEHLED------KGEKFRVPVNEHLSPA-AGTRCSV--------EEGRLP----TP-----------------------------
limpet             --------------GRTRQVKTAWQYDEPEEIYNGDSAEVDEEVETPVDED-YSNNNYRRGGRLP----TP-----------------------------
oyster             DYRQDDLSLMVQEGSERRAKS------RGRKKHRSRRARRREQEEASEVTP----DFPSDTGRIP----TP-----------------------------
aplysia            --------------EQKESVA------EQRRQLEETRQREDMKDDDDNDSE----GLVGKEGRLP----TP-----------------------------
Clustal Consensus

                            1210      1220      1230      1240      1250      1260      1270      1280      1290      1300
```

```
                   ....|....|....|....|....|....|....|....|....|....|....|....|....|....|....|....|....|....|....|....|
daphnia            WTVPLDTGKTFTVTQNI-RGGEIITSRPHSELKVSMTVRPPAPAVAAVHSPMIPIVSSSSAAAVTKPSEQQQQESQLNSGPAPDDPVPCNETEEETSKVA
silkworm           --------------RSA------SVAKDHFFD--------DSPVVKS----------------------------------------PPEPTRVKSP---
butterfly          --------------TKT------GTGLVNGAV--------EANGAGR-------------VNVTTPPARR-----------------SRSVSKVGIK---
bee                -----------QVHRKSFLATTAPPYRPLHHIKPSTTTTSTTTITTTTTTTTITTTTNTTSTSVKPPVKHSIHTS------------VHHPPTSSAAAAA
fly                -----------SASSAQ-QQ---QQQQQQQQMRKKDKDRSNSSCIGS-------------GSHVGAGSGS-----------------QANSVKHLQN---
mansoni            -----------------------PCRRYKADM---------KCTRDN-------------YVGILPAATK-----------------YNHTDEAYDS---
viverrini          -----------QLVRQN-DA---QALSTHFEN--------QLSST------------------------------------------SPDIEMEKHG---
sinensis           --------------QSTCSK---GGEKNNQSQ--------PVSQNDA-------------RTLSAHFDNQLSST-------------SPNIEMEKHG---
anemone            -----------GL----------GDVAIQHAQ--------GE----G-------------AVGIDPPRVS-----------------TREENSRPRV---
Trichoplax         -----------------------AGQGNGNDQ---------FEKLGS-------------NTIVER---------------------RNPIDNKAYE---
urchin             -----------TL-KES-EQQSHGSKRHHLDR--------TTPAKGG-------------ALLTSPASRRRATHVPKTTVTYPRQGELMKPSSRTYVARS
Saccoglossus       -----------RLTKKP-DA---SKQRHHLAR--------TTPAVGG-------------ALLSSPNIKRSSGVK------------KQSPSSKESGAKL
ciona              -----------QL-K-K-LG---GALRTHHDI--------TTPSRGG-------------ALLSSPPRPR-----------------RMISS--------
telata             -----------QG-RKR-SS---PYVRHHLDR--------TTPCAGG-------------ALLSTPPKAKVTEFKAIRGQEEITSDDRPASPERVSCTCG
Medaka             ------------L-K---AK---PVQRSRSDL--------GSPAAGG-------------TVLVGQQGNA-----------------DVSSPKKVQR---
tilapia            -----------EI-K---LK---PVQRTHHDL--------TTPAAGM-------------QTSFLHISVQ-----------------RCGSAVSM-----
stickleback        -----------RL---K-MR---PVQRTHHDL--------TTPATGG-------------AILVGKLSSD-----------------EASPNKPRRE---
molly              -----------RL---R-TR---PVQRTHHDL--------TTPATGG-------------AILVGKPTNR-----------------DDSSPIKQKQ---
platyfish          -----------RL-KTR------PVQRTHHDL--------TTPATGG-------------AILVGKPTNR-----------------DDSSPIKQKP---
mouse              -----------RL-R-E-L----GIQRTHHDL--------TTPAVGG-------------AVLVSPSKVK-----------------PPGLEQRRRA---
human              -----------KL-R-E-LG---GIQRTHHDL--------TTPAVGG-------------AVLVSPSKMK-----------------PPALEQRKRM---
cat                -----------KL-R-E-LG---GIQRTHHDL--------TTPAVGG-------------AVLVSPSKVK-----------------PPVPEQRKRV---
cattle             -----------KL-R-E-LG---GIQRTHHDL--------TTPAVGG-------------AVLVSPSKVK-----------------PPVIEQRKRI---
python             -----------QL---RALG---GIQRTHHDL--------TTPAAGG-------------AVLVSPQKVK-----------------HSSSLQIKKG---
chick              -----------KL-R-A-LG---RAQRTHHDL--------TTPAVGG-------------AVLVSPPKFK-----------------SSTSQQRMRS---
xenopus            -----------KL---KEFG---LSQRTHHDR--------TTPSTGG-------------ALLVSPMKEH-----------------LHTPEDKKKG---
shark              -----------KL-K-T-MV---APRRTHHDL--------TTPAVGG-------------AVLVSPYKIR-----------------SPSPHRKRNQAP-
gar                -----------KM---KTSG---VAQRTHHDL--------TTPATGG-------------AILVSPPKQK-----------------PSPQRVPRKSEPP
zebrafish          -----------KL-K-T-MQ---TVQRTHHDR--------TTPSIDF-------------NLHYIAKAAN------------------------------
tetra              -----------KL-K-T-IR---TVQRTHHDR--------TTPTIGG-------------VVLVSPPKTK-----------------NSSRTARRSEAP-
cavefish           -----------KL-K-T-IR---TVQRTHHDR--------TTPTIGG-------------VVLVSPPKTK-----------------NSSRTARRSEAP-
limpet             -----------HL-QKQ-DR---PVSRHHFDL--------TTPSVGG-------------ALLSSPPSVR-----------------SLPAKTRLSR---
oyster             -----------KM-R-S-KS---AGARHHLDR--------TTPVVGG-------------AMLSSPPRPE-----------------KKSPVKVSRSWCT
aplysia            -----------LL-Q-G-DS---SARRHHLDL--------TTPAVGG-------------AILTCPPSKS-----------------SKLIITSR-----
Clustal Consensus

                         1310      1320      1330      1340      1350      1360      1370      1380      1390      1400
                   ....|....|....|....|....|....|....|....|....|....|....|....|....|....|....|....|....|....|....|....|
daphnia            SQDETTGEQHPVEEDPVDQEKVAVAAEEETNPVEISSGEKEEEEEEEKEEVDCKAEESVEAEIKAQEEEEVEKETVKEKEAEEKSMDEIKEIRPADERQD
silkworm           ------------------------------------------------------------------------------------EQMNMRSPDPV-----
butterfly          -------LDDDIPNHRSASVAKDHFFDD--------------SPIVKSPPEPTRVKSP--------------------------EQMNMRSPDPVNWTV-
bee                RPDRVKDISRSHQGPNEAQAKTGRDQSAAAAAAAAVADPSKSSSDSRYASNQAATAVQPMTAEPQVNGDVTGGESSVASTPPSQSQAPVSATAAS-----
fly                -------KQKQQQLQQQQQQKQQQQQQPQQQQQKQQQKQQQQLDVAAAATAAAATSAP--------------------------PQNPTRPATIN-----
mansoni            ---------------------------------------------------ANHSLKS--------------------------ETDSIRLANSN-----
viverrini          ------------------------------------------------------------------------------------TAGQLSNIHRT-----
sinensis           ------------------------------------------------------------------------------------AASQPSHIHRT-----
anemone            -------EDQTLRGKGIGKAKDARVTHL-------RRLPTLHELNEPIDDRTRKLFTG---------------------------RHHVASDDRS-----
Trichoplax         ------------------------------------------------------------------------------------PKNGLNKEYKG-----
urchin             APVKAIIPGGITKGFEYDSDDDDDEDDVDLDRTLTRGQQTPPRNPTQQKQMGRFLHSS--------------------------PSAGRRTSDPH-----
Saccoglossus       QPASENGYRSDDEDSVPQLSRKMVQKSS--PKHSRYQADKVPCSCGADVKSHTKLIKS--------------------------PSAGVHTRDPD-----
```

```
ciona              --------------------------------------------------SSSPTLLS--------------------------KASSVCSVNNK-----
telata             -------VGSSLSPSPDEAPSQEKGVQT---------------------RTSSRLVFN--------------------------RDGSLVDPRKI-----
Medaka             --------------------------------------------------SSSLVSMT--------------------------TEAGIPCGMPV-----
tilapia            -------------------------------------------------------------------------------------AAGS--ETPA-----
stickleback        ------------------------------------------------------------------------------------------SSADQ-----
molly              ------------------------------------------------------KFAS--------------------------PEFGSEVGVNK-----
platyfish          ------------------------------------------------------KFAS--------------------------PEFGSEVSVNK-----
mouse              ---------------SSQDGLETLKKDI----------------TKKGKPRPMSLLTS--------------------------PAAGMKTVDPL-----
human              ---------------TSQDCLETSKNDF-----------------TKKESRAVSLLTS--------------------------PAAGIKTVDPL-----
cat                ---------------SSQDGLETAKNDF-----------------RKKESRAIALLTS--------------------------PAAGIKTVDPL-----
cattle             -------SSQD--------GLETLKNDF-------RKK----------ESRTISLLTS--------------------------PAAGIKTVDPL-----
python             -------SSEKQSLANSQVSREQLKRKT-------------------IRGEEKAVSNS--------------------------PAAGLETLDPL-----
chick              -------LGTDPSSVGQGASEASQRRSF-------RPGA---------KLKAVSLLTS--------------------------PPAGLGTVDPL-----
xenopus            -------YFVSSTPPKKAVCKKDIKTKL----------------IKDKELTGTPNTSP--------------------------PVAGIRTVDPL-----
shark              -------LGKSYSPYRCFSSGSPKKKLT----------------------KTESLQQS--------------------------PAAGIRTPDPI-----
gar                -------LGKPYSPYKHLARDSSPPQNP------------------SKAEERCSSQSP--------------------------PAAGLKTTDPI-----
zebrafish          ---------------------------------------------------GSLPRSS--------------------------PVAGLTTVDVL-----
tetra              -------LGKVHSPFRHLSHGSPPRAHS-------------------KAENGSLPRSS--------------------------PAAGLATIDPL-----
cavefish           -------LGKVHSPFRHLSHGSPPRAHS-------------------KAENGSLPRSS--------------------------PAAGLATIDPL-----
limpet             --------TQPIPPYDQDLNEDDEEEAR----------------MIGKTYNLIKNTPK--------------------------PTYGLPSRDTH-----
oyster             -------PQDVYDDEVSTVLSSDRFEPV-------VGQK----LAKTYDVKKGPLATT--------------------------PTFGLASKDTH-----
aplysia            -------SGDSYVTASDDLPVRGEQKKY----------------------NMDKLLTL--------------------------PAGGKPSPDTH-----
Clustal Consensus

                           1410      1420      1430      1440      1450      1460      1470      1480      1490      1500
                   ....|....|....|....|....|....|....|....|....|....|....|....|....|....|....|....|....|....|....|....|
daphnia            EKCSPVSDPLGAAIVLQGKGPLN-EDVPA----AIAPVESVTSIVPGTTLRRLEDPTPFLFSRPGTLGSLNAGQPMASSTSLS----DTKPKAPRYRVLE
silkworm           --------------------------NWT-------------VPLDTGKTFTVTQNVK---------------NDESIKRPSS----EFKSGSIA-----
butterfly          --------------------PLDTGKTFT----VTQNVKSEIAPIHHQQMSPIRSLKKSESPTSLS-------KRTDKTPTTP----ISLTPIDE-----
bee                --------------------PWI-DDEPV----VKSPPEPTRVKSPEQMIMRSPEPVNWTVPLDTGKTFTVTQNVREEPLTRPHSEAKSWAPSS------
fly                --------------------IKRLTVPGQDRKLAENEGEDGRETAISISSCSPQPPVP---EVPEE-------PLVKSPPEPT----RVKSPEQI-----
mansoni            --------------------EPRTNNSYI----------------------------------------------------------KQWSNDSK-----
viverrini          --------------------SQQGNSLSP-----------------------------------------------VSTKSIM----SSATPISE-----
sinensis           --------------------SRKGNSLSP-----------------------------------------------VSTKSIM----SSATPISE-----
anemone            --------------------PRN-DSPPT----RQPPEQQRVDSKTRKLFTGRPLETSDSTELELS-------VDTSASNSTT----HSSLPPPR-----
Trichoplax         --------------------NLKNVDTYS---------KAKTTVGFANHKNEATKENR---------------NLTSDSNIRP----PKSSNLRR-----
urchin             --------------------PLN-EELISPGFQRHSERFFETSPGMPTIPNKIEPPKTWAAPWAGL-------KTAPPAGKGP----LERPHSGR-----
Saccoglossus       --------------------PIE-ENYPTSPFQRHSQNRFETGPVRLPEESSPPRPVD---SRPLKTWAGHPVDQPRPKVTPK----KDSRESDQ-----
ciona              --------------------TFL-EDSKS------------------------KRSPS---PGCRM-------VRTDQGLPRR----LQASLRAP-----
telata             --------------------QVNLEEIFA----DRKPVERKLTDDSVEGSDGLPTFGM-----PTRDTHELLDEEASTDRPLA----THYVQTPQ-----
Medaka             --------------------KRK-E-AWP----EKV-------STHQHEPFFSPSPKP---NS-------------QASSSPP----LAPPPLHA-----
tilapia            --------------------KRK-E-AWS----DNIAVSYS--PAHDHKSASSPSFKP---TRMKQ-------TLSPAAAPPP----LVPAPLHG-----
stickleback        --------------------PVKLKEAWS----DNSPAFAHTGPSLDHKPATSPASKP---IRTKQ-------T--SPMAPRP----LAPPPPHC-----
molly              --------------------PVKLKEAWP----ENSPVSLSPPPTNQQTPSPQPQPIR---TKQAP-------P--SPAAPPP----LGSPPQHG-----
platyfish          --------------------PVKLKEAWP----ENTPMSLNPPPTNKQTPSPQPQPIRTKQAPP------------SPVAPPP----LGSPPQHG-----
mouse              --------------------PLR-EDCEA-----NVLRFAEGTLPVSKILDRQPSTPG------------------QLPPCAP----PYCHPSSR-----
human              --------------------PLR-EDSED-----NIHKFAEATLP----VSKIPKYPT---NPPGQ---------LPSPPHVP----SYWHPSRR-----
cat                --------------------PLR-EDSEI-----SIPRRAEVALP----VSKILEYPA---NTPGQ-------S--PSPPYAP----SYWHPSRR-----
cattle             --------------------PLR-EDSEP-----NIPKFVETTLP----VSKMPEYPP---NTPGK-------S--ASPPCAT----SYWHPSRR-----
python             --------------------PLRKDQCTNQGTNQDTSGTRNYETSVHSEQMPTVSETN------------------LAKGSSL----PYWSPLCR-----
chick              --------------------PLR-QDQWH-----PNGVSGETVAI----NSAYQERSS---TPPVL-------K--STKNRPM----PCWSPSPR-----
```

```
xenopus             --------------------PLR-EDPWP-----------------IAYISNTSSPPRVTVPASKS-------PEPRAYVSMS----PLWNTSCR-----
shark               --------------------PLR-EECWI--------------PNTPGCLKTIPSVLALLKPDCTF-------DEKSASVPAS----PEKGYLHR-----
gar                 --------------------PLR-VDSWP----SDSPHKAQTSPT-------AAKPFR---------------KHSASLAPVP----AGRTPACR-----
zebrafish           --------------------PMR-EDVWS--------------------DEEVTDYRSLKPPKPSR-------KQQSSQRHKA----NAAPPANR-----
tetra               --------------------PLR-DDTWA----RDSP---------------EPKPVR---TPQRR-------T--LSATLIA----TSSPRANR-----
cavefish            --------------------PLR-DDTWA----RDSP---------------EPKPVR---TPQRR-------T--LSATLIA----TSSPRANR-----
limpet              --------------------YLL-DEEAS----TDRPMRTQFLQTEVFDDGDTESTVT---EGEIQ-------TTQPKIVDEP----RYKRQSPS-----
oyster              --------------------FLR-DDEVS----VDKPLETVFVDS-------PAKVRM---TGNVS-------S--GDEKPAR----KSK-KPTK-----
aplysia             --------------------ALR-DDVAE----SDQALVTQY----------IVSPPL---PGSEE-------DKENKGQRKK----KSKGQKPG-----
Clustal Consensus

                          1510      1520      1530      1540      1550      1560      1570      1580      1590      1600
                    ....|....|....|....|....|....|....|....|....|....|....|....|....|....|....|....|....|....|....|....|
daphnia             APGMMSD--PV---VHEPSYEPHPL---------------------QSSSSTAPMMTSSSPYKGRVLEAPSSPP-------KSSTSSSAAAFTSSPSTDS
silkworm            EEGVIVK--PA---------------------------------------------------------------------------------------GT
butterfly           ----TKA--LD---------LNKVN---------------------GVNGLNSNQLTPETPVQEHLAEKEVIKK-------STEATQVAPGVVDTTVVNE
bee                 VPSAPQSAPPELAAQHKSQHSQHSGYKSPESESVSIGSFSGLNGHKDMDSERDSPLP--SNIQTGGTPTHVDDE-------TGGIEEESKEQRSEPSIKS
fly                 ---IMRS--PD----------PVNW---------------------TVPLDTGKTFT--VTQNVKDGENYSRPQ-------SEIKASTPV--------EK
mansoni             NHASKRQ--NH---------WFHNE----------------------------------YERKQRTVDETESLT-------S--CSSLSE--------KS
viverrini           ---------PT-----------------------------------N------------YTNEQRNHAVNDNSH-------QR---------------RC
sinensis            ---------PA-----------------------------------S------------YTNKQRNYAVNDSNN-------QR---------------RI
anemone             VDSRTKH--IH------------------------------------MGRPAGGRAT--STESESYSSGDELSS-------SLSSDALSF--------EA
Trichoplax          KFGQNATTIVK---------RNENG---------------------NVAGNENSTDRGYYSNSHRNTGDNNPHDGNIAEISDR-ISKLKNRLLDDRESVS
urchin              IGGRPQTAVPQ---------TREAA---------------------SQQVQRLHSFM--SPRSNGLESPDD-------------WNLTPP--------RG
Saccoglossus        PPSYLRK--PAEAWSVPLEPSTRAA---------------------QYSRPDSGFIT--PPPRPEARLDLQDDE-------DR-WSVKST--------GT
ciona               IKGALKS--QE---------FQHCG---------------------KNSRPMTA-------------------V-------DR-LSMVSN--------RS
telata              NARLARQKFPE---------ADIPS---------------------TIDENMAQTFNKNKLLPGEVCLDDIPNW-------RRDEDALSTSV------RS
Medaka              IQGSMRH--PD---------FQHNG---------------------E----------------------HR--Q-------NG-LVMSGP--------PD
tilapia             LQGTLRH--PD---------FQHNG---------------------ELG---LRFRE--RPCPGGGCCSDE--D-------DR-LSVMSW--------RS
stickleback         IRGTLRN--AD---------FQHNG---------------------ELGLRFRE-----LRCPGRGSCSHE--D-------DR-LSVMSW--------RS
molly               IQGTLRH--PD---------FQHNG---------------------EL--------------------------------------------------QT
platyfish           IQGMLRH--PD---------FQHNG---------------------DLGLRFRE-----LPCSRGGCGSDE--D-------DR-LSVMSW--------RS
mouse               IQGRLRD--PE---------FQHNN---------------------A----------------------------------DR-LSEISA--------RS
human               IQGSLRD--PE---------FQHN-----------------------VGKARMNNLQ--LPQHEAFNDED---E-------DR-LSEISA--------RS
cat                 IQGSLRD--PE---------FQHN-----------------------VGKARMNNFQ--LPQHEVFNDED---E-------DR-LSEISA--------RS
cattle              IQGSLRD--PE---------FQH-----------------------NVGKSRMNNFQ--LHQHEA-FNDED--E-------DR-LSEISA--------RS
python              IQGTLRD--PE---------FQHNG---------------------NIASPKRSWLQ--LPLQERNYNDEDD-D-------DR-LSQLSA--------RS
chick               IQGTLKD--PE---------FQHNG---------------------NVGNPKMGSFQ--LPLQER-NYNNE--D-------DR-LSQISA--------RS
xenopus             IQGALRD--PE---------FQHNG---------------------NFGS---------PGLHRTAYSDDCSID-------DDRLSQISE--------RS
shark               IQGTLRN--PE---------FQHNG---------------------NTGGPRSSILR--MQSTDSAAAADE--D-------DR-LSQISA--------RS
gar                 IQGALRD--PE---------FQHNG---------------------NLGDLRPGLFA--FSSNDSDISDN---D-------DR-MSQISS--------RS
zebrafish           IQGTMRN--AE---------FQHNG---------------------NLGIFRPELFV--LPQSDSALSDN---D-------DK-MSQISS--------RS
tetra               IQGTLRN--PE---------FQHNG---------------------NLGLHRPDQFV--FLSSDSGVSDN---D-------DR-MSQISS--------RS
cavefish            IQGTLRN--PE---------FQHNG---------------------NLGLHRPDQFV--FLSSDSGVSDN---D-------DR-MSQISS--------RS
limpet              --APAMD--PI---------YENFG---------------------QTYNKAYVPPI--YSYNDVENYYRDDDD-------NESVLSLSV--------RS
oyster              FE--AQR--TI---------KEGYG---------------------QTYNK---PMV--WGIDGGMTIPRIEPD-------DD-ALSLSV--------RS
aplysia             SLTTIKE---------------GYG---------------------KTYQPKPF-----LDLDDIKAGTLDKKD-------DD-VLSVSL--------MS
Clustal Consensus

                          1610      1620      1630      1640      1650      1660      1670      1680      1690      1700
                    ....|....|....|....|....|....|....|....|....|....|....|....|....|....|....|....|....|....|....|....|
```

```
daphnia            LTSSRSL------------------------------------------------------------------------------------A-SDVLEKA
silkworm           -TNTATA---------------------------------------------------------------------------------------------
butterfly          TPTGGLT------------------------------------------------------------------------------------A-SEVLDRA
bee                VAGTSLRCLEDPSFEYERKKEGSQPVTTAAMTAPSSTTTPQQGYRILEAESPQGGAIDSGGAGSVGGIGSSGGGYHVLEAPTVVPGSAQRSAASEVLEKA
fly                PPPPPQS------------------------------------------------------------------------------------APPQLTEQA
mansoni            MESSARL------------------------------------------------------------------------------------A-HETLERA
viverrini          TLPSGDY------------------------------------------------------------------------------------L-CHPYAEN
sinensis           IHPSGDY------------------------------------------------------------------------------------L-CHPYAES
anemone            MKKRKDL------------------------------------------------------------------------------------A-TKTLQRA
Trichoplax         -SMASSY------------------------------------------------------------------------------------A-SDVLERS
urchin             GGGGGET------------------------------------------------------------------------------------AMSSPYQPC
Saccoglossus       VSSSCSL------------------------------------------------------------------------------------A-SEVLERA
ciona              ----NAS------------------------------------------------------------------------------------A-AKLLERS
telata             VASSCSV------------------------------------------------------------------------------------A-SEIFERA
Medaka             -SAP------------------------------------------------------------------------------------------------
tilapia            -AASCST------------------------------------------------------------------------------------A-SAVLERA
stickleback        -AASCSA------------------------------------------------------------------------------------A-SVVLERA
molly              -PVLCLH------------------------------------------------------------------------------------L-SFH----
platyfish          -AASCSA------------------------------------------------------------------------------------A-SAVLERA
mouse              -AVSSLR------------------------------------------------------------------------------------A-FQTLARA
human              -AASSLR------------------------------------------------------------------------------------A-FQTLARA
cat                -AASSLQ------------------------------------------------------------------------------------A-FQTLARA
cattle             -AASSLQ------------------------------------------------------------------------------------A-FQTLARA
python             -AVSGSF------------------------------------------------------------------------------------A-SQVLERA
chick              -AASSSL------------------------------------------------------------------------------------A-SQILERA
xenopus            -AATGSL------------------------------------------------------------------------------------A-SQVLERA
shark              -AASSTL------------------------------------------------------------------------------------A-SQVLERA
gar                -AASSSL------------------------------------------------------------------------------------A-SEILERA
zebrafish          -AASCSM------------------------------------------------------------------------------------A-SEVLGRA
tetra              -AASCSM------------------------------------------------------------------------------------A-SEVLDRA
cavefish           -AASCSM------------------------------------------------------------------------------------A-SEVLDRA
limpet             IASSCSY------------------------------------------------------------------------------------A-SEILERS
oyster             IASSCSL------------------------------------------------------------------------------------A-SEVYERA
aplysia            VASSGSL------------------------------------------------------------------------------------A-SEVLERA
Clustal Consensus

                           1710      1720      1730
                   ....|....|....|....|....|....|....
daphnia            RTRFDRFWTKKESDK-------------------
silkworm           ----------------------------------
butterfly          RTRFDKFWGKKEDDV-------------------
bee                RNRFDKFWGKGSGSEN------------------
fly                KMDGKPLSTLQ-----------------------
mansoni            QKQHERILREQKQYNENIYSPNAYSECECKRIEI
viverrini          HEYRS--WDYI-----------------------
sinensis           QEYRS--WNYI-----------------------
anemone            RNRGARF---------------------------
Trichoplax         KQRKENFWGRK-----------------------
urchin             PMHPAAV---------------------------
Saccoglossus       RRRRDEFWGKQPSVQTK-----------------
ciona              MQR-QDFWANK-----------------------
```

```
telata             KKRRDLFWAPNGENSDVSHRHRIMLLVGTSTHGS
Medaka             ----------------------------------
tilapia            QKRREDFWGKR-----------------------
stickleback        QKRREHFWGKR-----------------------
molly              ----------------------------------
platyfish          QKRREDFWGKR-----------------------
mouse              QKRKENFWGKP-----------------------
human              KKRKENFWGKT-----------------------
cat                QKRKENFWGKT-----------------------
cattle             QKRKENFWGKT-----------------------
python             QRRKENFWGKM-----------------------
chick              QKRKEDFWGKT-----------------------
xenopus            QRRKEDFWGKK-----------------------
shark              QMRRDSFWGKNPRNEQ------------------
gar                QRRKENFWGKS-----------------------
zebrafish          QRRKQEFWGKS-----------------------
tetra              QRRKEDFWGKT-----------------------
cavefish           QRRKEDFWGKT-----------------------
limpet             KDRRDNFWNNNTRVPTK-----------------
oyster             QKRTQEFWGKDG-IASR-----------------
aplysia            RKR-QEFWNKKDS---------------------
Clustal Consensus
```
