# Supplementary material for: Molecular Evolution of MDM1, a “Duplication-Resistant” Gene in Vertebrates
Source: PLoS One. 2016 Sep 22;11(9):e0163229. doi: 10.1371/journal.pone.0163229 (PMC5033493; doi:10.1371/journal.pone.0163229)
Supplement: S1 Table — (DOCX) [file pone.0163229.s004.docx]

| Common name | Species | Sequence ID |
| --- | --- | --- |
| Acorn worm | *Saccoglossus kowalevskii* | XP_006816368 |
| Amazon molly | *Poecilia formosa* | ENSPFOT00000023992 |
| Blood fluke | *Schistosoma mansoni* | CCD75536 |
| Burmese python | *Python bivittatus* | XP_007423469 |
| California sea hare | *Aplysia californica* | XP_005097852 |
| Cat | *Felis catus* | XP_003989070 |
| Cattle | *Bos mutus* | XP_005909108 |
| Cave fish | *Astyanax mexicanus* | ENSAMXT00000004259 |
| Chicken | *Gallus gallus* | ENSGALT00000016130 |
| Common water flea | *Daphnia pulex* | EFX81895 |
| Domestic silkworm | *Bombyx mori* | XP_004923605 |
| Elephant shark | *Callorhinchus milii* | XP_007903187 |
| Human | *Homo Saoiens* | NP_059136 |
| Fruit fly | *Drosophila Melanogaster* | NP_001097706 |
| Honey bee | *Apis mellifera* | XP_006570794 |
| Japanese medaka | *Oryzias latipes* | XP_004083352 |
| Liver fluke | *Opisthorchis viverrini* | KER25769 |
| Liver fluke | *Clonorchis sinensis* | GAA35926 |
| Mexican tetra | *Astyanax mexicanus* | XP_007232347 |
| Monarch butterfly | *Danaus plexippus* | EHJ76871 |
| Mouse | *Mus musculus domesticus* | AAA39511 |
| Nile tilapia | *Oreochromis niloticus* | ENSONIT00000022736 |
| Owl limpet | *Lottia gigantea* | XP_009049003 |
| Pacific oyster | *Crassostrea gigas* | EKC42562 |
| Platyfish | *Xiphophorus maculatus* | ENSXMAT00000006560 |
| Polychaete annelid worm | *Capitella teleta* | ELU03143 |
| Purple sea urchin | *Strongylocentrotus purpuratus* | XP_003723432 |
| Spotted gar | *Lepisosteus oculatus* | ENSLOCT00000019971 |
| Starlet sea anemone | *Nematostella vectensis* | XP_001631049 |
| Stickleback | *Gasterosteus aculeatus* | ENSGACT00000000919 |
| Trichoplax | *Trichoplax adhaerens* | XP_002116097 |
| Vase tunicate | *Ciona intestinalis* | XP_009858197 |
| Western clawed frog | *Xenopus (Silurana) tropicalis* | NP_001123739 |
| Zebrafish | *Danio rerio* | NP_001038285 |
| Lancelet | *Branchiostoma floridae* | XP_002601763 |
